# A High-Throughput Microtiter Plate Based Method for the Determination of Peracetic Acid and Hydrogen Peroxide

**DOI:** 10.1371/journal.pone.0079218

**Published:** 2013-11-18

**Authors:** Karson S. Putt, Randall B. Pugh

**Affiliations:** Johnson & Johnson Vision Care, Inc., Jacksonville, Florida, United States of America; Concordia University Wisconsin, United States of America

## Abstract

Peracetic acid is gaining usage in numerous industries who have found a myriad of uses for its antimicrobial activity. However, rapid high throughput quantitation methods for peracetic acid and hydrogen peroxide are lacking. Herein, we describe the development of a high-throughput microtiter plate based assay based upon the well known and trusted titration chemical reactions. The adaptation of these titration chemistries to rapid plate based absorbance methods for the sequential determination of hydrogen peroxide specifically and the total amount of peroxides present in solution are described. The results of these methods were compared to those of a standard titration and found to be in good agreement. Additionally, the utility of the developed method is demonstrated through the generation of degradation curves of both peracetic acid and hydrogen peroxide in a mixed solution.

## Introduction

The advantages of using the antimicrobial properties of peracetic acid increasingly have been recognized and are gaining usage in a number of industries including the treatment of wastewater [1], usage in hospitals, medical clinics and pharmacies [2], [3], dental practices [4], aquaculture [5], the dairy industry [6] and food preparation [7], such as seeds and sprouts [8], fruits and vegetables [9] and poultry and other fowl [10]. Additionally, other properties of peracetic acid have found use in other industries, such as in the treatment of bioenergy crops [11] where it is used to remove difficult to digest components and in the bleaching of paper products [12]. As the usage of peracetic acid gains greater acceptance, the need for accurate and higher throughput methods to quantitate the concentration of peracetic acid are required.

Peracetic acid is commercially available in many different concentrations and mixtures, however nearly all peracetic acid preparations contain hydrogen peroxide. Hydrogen peroxide is often a reactant used in the synthesis of peracetic acid and can also be a byproduct of peracetic acid degradation. As these two different peroxides have different chemical properties and biological effects, the concentration of both the peracetic acid and the hydrogen peroxide often must be determined. Classically, peracetic acid and hydrogen peroxide have been determined using reduction/oxidation titrations followed by the formation of iodine from iodide [13]. In the 100 years since this initial work was performed, many other methods have been developed to specifically quantitate peracetic acid.

Methods for the separation, derivatization and detection of these peroxides, such as gas chromatography [14], [15], [16] and HPLC [17], [18], [19] have been developed. While these chromatographic methods are able to quantitate both peracetic acid and hydrogen peroxide, like nearly all chromatographic methods, large numbers of solutions cannot be rapidly processed. In addition to these methods, potentiometric and amperometric methods have also been developed [20], [21], [22], [23]. However, most of the electrical signal based methods can only determine peracetic acid and not hydrogen peroxide; have limits in sensitivity, dynamic range or specificity; may require frequent calibrations and require specialized or custom made materials. Optical cuvette based methods employing enzymes such as catalase also have been developed [24]. However, these methods rely on enzyme kinetics and involve continuously measuring the sample until a short lived absorbance minimum is reached.

Additionally, one of the main challenges for an accurate measurement using these techniques is the creation of a standard curve from known materials. While pure hydrogen peroxide can be readily obtained, pure distilled peracetic acid is highly reactive and can be difficult to safely produce. The breakdown of both these peroxides, especially the peracetic acid can also cause difficulties in maintaining accurate calibration curves. Even if these issues could be overcome, none of the methods listed above can rapidly quantitate both peracetic acid and hydrogen peroxide concentrations in many different samples in a short period of time.

Currently, the greatest throughput microtiter plate based method that has been developed utilizes the reaction between peracetic acid and 2,2A-Azino-bis (3-ethylbenzothiazoline)-6-sulfonate [25]. While this method does allow for the rapid determination of peracetic acid, it does not quantitate the levels of hydrogen peroxide. Even with all of these methods and their improvements, titrations are still the most widely regarded and commonly utilized industrial method to quantitate peracetic acid and hydrogen peroxide concentrations [25]. Clearly there is a need for a rapid and higher throughput method to accurately determine the concentration of peracetic acid and hydrogen peroxide in solution. In an effort to develop such a rapid and higher throughput method, the chemistry relied upon in the peracetic acid and hydrogen peroxide titrations were explored and modified to create a microtiter plate based absorbance method.

## Materials and Methods

### Materials

Cerium (III) sulfate, cerium (IV) sulfate, 0.5 M iodine solution, potassium iodide, sodium hydroxide, 0.1 M sodium thiosulfate solution, 98% sulfuric acid, hydrogen peroxide, peracetic acid/hydrogen peroxide mixture and starch solution were purchased from Sigma-Aldrich (St. Louis, MO). Ferroin indicator was purchased from Taylor Technolgies (Sparks, MD). Dulbecco's phosphate buffered saline solution (PBS) was purchased from Mediatech (Manassas, VA). SpectraMax 384Plus absorbance plate reader was purchased from Molecular Devices (Sunnyvale, CA). Flat bottom 96-well polystyrene plates (Corning #3598), 50 mL polypropylene tubes (BD #352098), 15 mL polypropylene tubes (BD #352099) and all other materials were purchased from VWR (Atlanta, GA). TableCurve2D was purchased from Systat Software (San Jose, CA).

### Absorbance spectra

200 µL of various concentrations of the compounds were added in quadruplicate to the wells of a Corning 96-well flat-bottom plate. The absorbance was read between 300 and 900 nm every 2 nm on a Molecular Devices SpectraMax 384Plus. The average absorbance from a DI water blank was subtracted to yield the final absorbance curve.

### Cerium (IV) standard curve

100 µL of a 10 to 100,000 µM solution of cerium (IV) sulfate in 10% (v/v) sulfuric acid/water was added in quadruplicate to the wells of a 96-well plate. 100 µL of deionized water was added to each well containing the cerium (IV) sulfate solution. 200 µL of deionized water was added to wells to serve as a blank. The absorbance of the plate was read at 300 to 500 nm every 25 nm. The values of the blank wells were subtracted and the linear range then was determined for each wavelength.

### Iodine standard curve

100 µL of a 10 to 100,000 µM solution of iodine was added in quadruplicate to the wells of a 96-well plate. 50 µL of deionized water followed by 50 µL of a 3% (v/v) sulfuric acid/water solution were added to each well containing the iodine solution. 200 µL of deionized water was added to wells to serve as a blank. The absorbance of the plate was read at 300 to 600 nm every 25 nm. The values of the blank wells were subtracted and the linear range then was determined for each wavelength.

### Peracetic acid/hydrogen peroxide determination via titration

A volume of peracetic acid/hydrogen peroxide was weighed in a 250 mL beaker. 50 mL of a 1 M ice-cooled sulfuric acid/water solution was added to the beaker and the beaker was placed on ice. 3 drops of ferroin indicator were added and the solution was titrated with various concentrations of cerium (IV) sulfate until the disappearance of the salmon color and the appearance of a light blue color. 10 mL of a 2.5 M potassium iodide solution was added to form a dark brownish-red color. The solution was then titrated with various concentrations of sodium thiosulfate. When the solution lightened to a brown-yellow, 1 mL of starch solution was added to produce a dark purple color. Additional sodium thiosulfate was added until the solution turned a salmon color. The volumes and molarity of the cerium (IV) sulfate and sodium thiosulfate were used to calculate the concentration of hydrogen peroxide and peracetic acid respectively.

### Hydrogen peroxide determination via microtiter plate based method

100 µL of a sample solution was added to the wells of a 96-well plate. 200 µL of deionized water was added to wells to serve as a blank. 100 µL of a 10 or 100 mM solution of cerium (IV) sulfate in 10% (v/v) sulfuric acid/water was added to each sample well and the absorbance of the plate immediately was read at 300, 400 and 450 nm. The DI water blank's absorbance was subtracted from the sample's absorbance and concentration of remaining cerium (IV) sulfate was then determined using the standard curve generated as described above. The amount of cerium (IV) sulfate consumed in the reaction was calculated, which is equivalent to twice the amount of hydrogen peroxide present in the sample.

### Total peroxide determination via microtiter plate based method

100 µL of a sample solution was added to the wells of a 96-well plate. 200 µL of deionized water was added to wells to serve as a blank. 50 µL of a 3% (v/v) sulfuric acid/water solution were added to each well containing the iodine solution. 50 µL of a 250 mM potassium iodide solution was added to each sample well. The absorbance was read immediately at 350, 450 and 550 nm. The DI water blank's absorbance was subtracted from the sample's absorbance and the concentration of iodine formed was determined using the standard curves generated as described above. The amount of iodine formed is equivalent to the amount of total peroxide present in the sample. The concentration of peracetic acid can be calculated by subtracting the concentration of hydrogen peroxide from the amount of total peroxide present in the sample.

### Effect of sulfuric acid concentration on total peroxide determination

100 µL of a 15 mM total peroxide solution or 100 µL DI water was added to the wells of a 96-well plate. 200 µL of deionized water was added to wells to serve as a blank. 50 µL of various concentrations of sulfuric acid were added to each well, except the blank wells. 50 µL of a 250 mM potassium iodide solution was added to each well, except the blank wells. The absorbance was read at 550 nm every minute for 60 minutes. The DI water blank's absorbance was subtracted from all other absorbance values. The concentration of iodine formed was determined using the standard curve generated as described above.

### Peracetic acid/hydrogen peroxide degradation

A commercially available preparation of peracetic acid/hydrogen peroxide in acetic acid was diluted in PBS to yield a final concentration of ∼150 mM peracetic acid and ∼45 mM hydrogen peroxide. Sodium hydroxide was added until the pH reached 7.5. Various volumes of solution were sampled at various times and the concentrations of peracetic acid and hydrogen peroxide were determined via titration as described above. Additionally, 100 µL of sample was added in quadruplicate to the wells of two 96-well plates and the amount of hydrogen peroxide or total peroxides were determined as described above.

## Results and Discussion

### Identification of quantifiable chemical reaction species

To create an assay amenable for the rapid determination of both peracetic acid and hydrogen peroxide in numerous mixed solutions, a microtiter plate based spectral method was desired. To create such a method, the reactions utilized in various published titration methods were explored. In the modified titration protocol of Greenspan and MacKeller [26], cerium (IV) sulfate is reacted specifically with hydrogen peroxide followed by the addition of potassium iodide which is converted into iodine by the remaining peracetic acid.

In order to determine if the reactants and/or products could be directly observed without the use of indicator solutions, such as the ferroin and starch solutions utilized in the titration, the absorbance spectra of cerium (III) sulfate, cerium (IV) sulfate, potassium iodide, iodine, sulfuric acid and peracetic acid/hydrogen peroxide solution were measured (see Figures S1 through S6). Most of these chemicals are clear or white and unsurprisingly did not have any significant absorption in the visible spectrum. However, the cerium (IV) sulfate and iodine did exhibit strong absorption in the blue region, giving rise to their yellow color. Unfortunately, since both of these chemical species absorb similar wavelengths of light, they could not be quantitated together in a single reaction mixture. Therefore, a two reaction strategy was employed to determine the concentration of both peroxides.

### Selective Hydrogen Peroxide Quantitation

**Figure 1:**
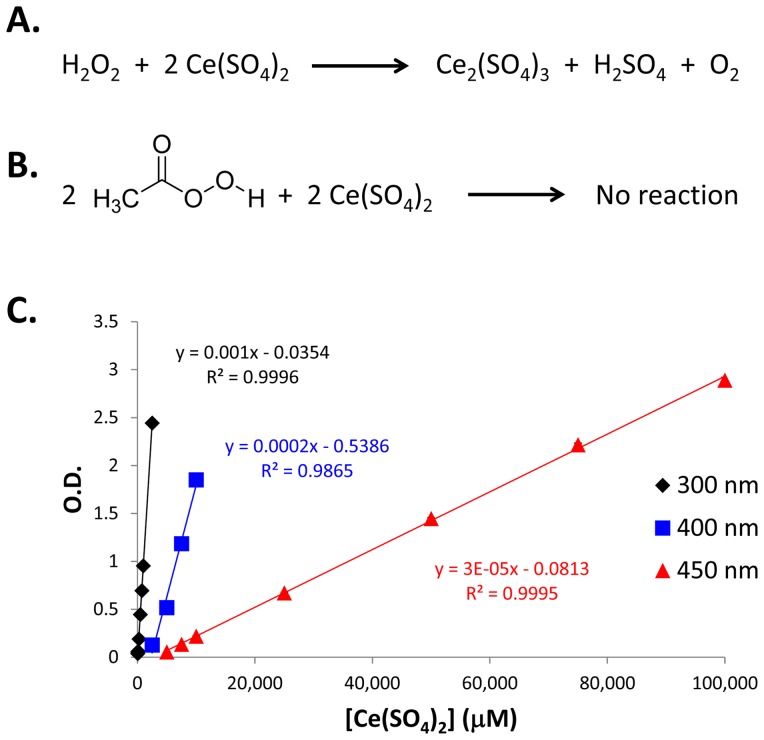
Overview of selective hydrogen peroxide quantitation. A.) Chemical reaction between hydrogen peroxide and cerium (IV) sulfate. B.) Chemical reaction between peracetic acid and cerium (IV) sulfate. C.) Multiple wavelength cerium (IV) sulfate standard curve (n = 4). Error bars represent standard deviation and are typically smaller than the plotted marker.

As shown in Figure 1A and B, the reaction of the colored cerium (IV) sulfate to the colorless cerium (III) sulfate proceeds in the presence of hydrogen peroxide, but not in the presence of peracetic acid. The selectivity of this reaction can therefore be used to specifically quantitate the concentration of hydrogen peroxide in solution. To accomplish this, a standard curve of cerium (IV) sulfate was established. First, 100 µL of various concentrations of cerium (IV) sulfate and 100 µL of DI water were added to the wells of a 96-well plate. The absorbance of cerium (IV) sulfate can be measured at multiple wavelengths depending upon the concentration of interest. Multiple standard curves were generated at various wavelengths (see Figures S7 through S15) and three wavelengths were chosen that spanned a low, medium and high concentration range (Figure 1C). At 300, 400 and 450 nm, a 50 to 2500 µM, 2500 to 10,000 µM and 5000 to 100,000 µM cerium (IV) sulfate solution could be quantitated respectively.

Since cerium (IV) sulfate and hydrogen peroxide are the reactants, the amount of cerium (IV) sulfate consumed is directly proportional, by a factor of two, to the amount of hydrogen peroxide initially present. Utilizing this relationship, a simple microtiter plate method was developed based upon the absorbance of cerium (IV) sulfate. First, 100 µL of sample or DI water is added to the wells of a 96-well plate, followed by the addition of a solution of cerium (IV) sulfate. The concentration of the cerium (IV) sulfate solution added can be adjusted based upon the expected values of hydrogen peroxide in the sample. As an additional note, the cerium (IV) sulfate solution should be contain at least a sulfuric acid concentration of 10% (v/v) as a lower concentration may cause the cerium (IV) sulfate to crash out of solution upon addition to the aqueous sample, obfuscating the absorbance reading.

The reaction between cerium (IV) sulfate and hydrogen peroxide is immediate, often with the visible evolution of oxygen at high concentration of hydrogen peroxide. The absorbance of the plate is read immediately and the starting concentration of the cerium (IV) sulfate solution, from the DI water wells and the ending concentration, from each sample well is calculated using the standard curve generated above. The final cerium (IV) sulfate concentration is subtracted from the initial to determine the amount consumed in the reaction. Due to the stoichiometry of the reaction, the amount of cerium (IV) sulfate consumed is equal to twice the concentration of hydrogen peroxide.

While this reaction is highly selective for hydrogen peroxide, as the hydrogen peroxide is consumed the equilibrium reaction between it and peracetic acid is shifted towards the formation of hydrogen peroxide from the breakdown peracetic acid into acetic acid. This additional hydrogen peroxide then can react with the cerium (IV) sulfate causing a false increase in concentration as time passes. The possibility also exists of a very slow reaction between the peracetic acid and the cerium (IV) sulfate as well. Because of these confounding factors, the plate absorbance should be read immediately as the false increase in hydrogen peroxide concentration, as measured by the decrease in absorbance was determined to be ∼2% per minute (see Figures S16).

**Figure 2:**
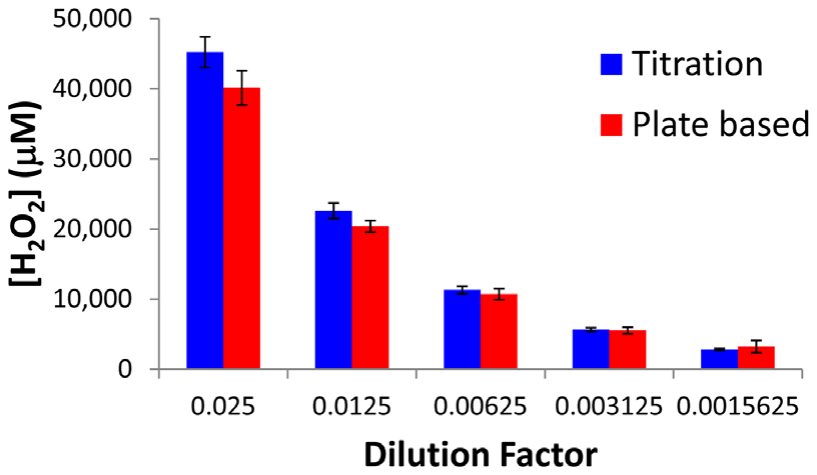
Comparison of hydrogen peroxide quantitation. The amount of hydrogen peroxide in various dilutions from a stock peracetic acid/hydrogen peroxide solution were determined via titration or the microtiter plate based method (n = 3, error bars represent standard deviation).

To compare the results obtained from the plate based hydrogen peroxide detection to those obtained from a titration, several dilutions of a peracetic acid/hydrogen peroxide solution were made and the concentration of hydrogen peroxide was determined by both methods. As can be seen in Figure 2, the two methods show good agreement generally being within the error of one another.

### Total Peroxide Quantitation

**Figure 3:**
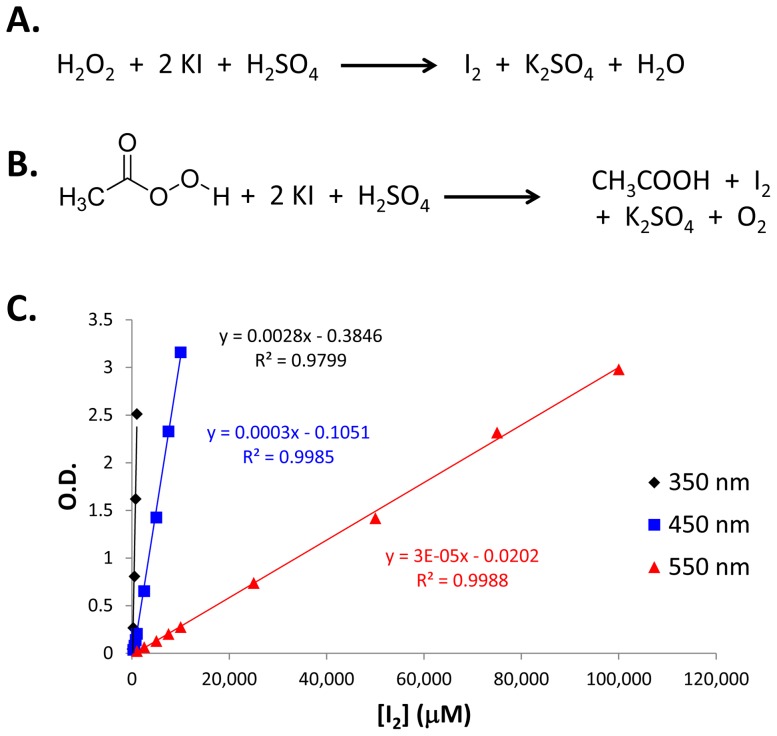
Overview of general peroxide quantitation. A.) Chemical reaction between hydrogen peroxide and potassium iodide. B.) Chemical reaction between peracetic acid and potassium iodide. C.) Multiple wavelength iodine standard curve (n = 4). Error bars represent standard deviation and are typically smaller than the plotted marker.

As shown in Figure 3A and B, the reaction of the colorless potassium iodide to the highly colored iodine proceeds in the presence of both hydrogen peroxide and peracetic acid, unlike the specific reaction of cerium (IV) sulfate. Utilizing the potassium iodide reaction, the total amount of peroxides present can be determined. To accomplish this, a standard curve of iodine was established. First, 100 µL of DI water, 50 µL of 3% sulfuric acid and 50 µL of various concentrations of iodine were added to the wells of a 96-well plate. Similar to the method above, the absorbance of iodine can be measured at multiple wavelengths depending upon the concentration of interest. Multiple standard curves were generated at various wavelengths (see Figures S17 through S27) and again three wavelengths were chosen that spanned a low, medium and high concentration range (Figure 3C). At 350, 450 and 550 nm, a 100 to 1000 µM, 250 to 10,000 µM and 250 to 100,000 µM iodine could be quantitated respectively.

**Figure 4:**
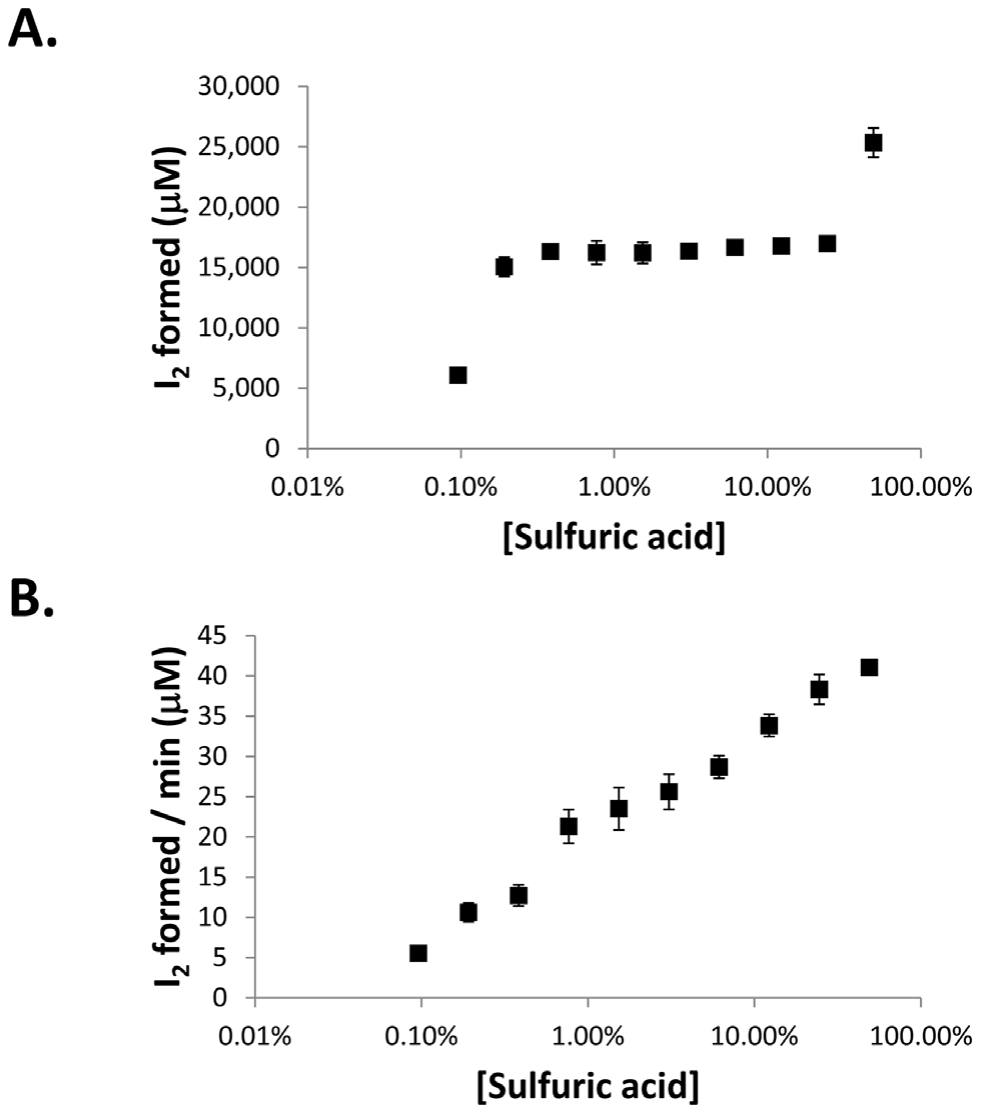
Effect of sulfuric acid concentration on peroxide – potassium iodide reaction. A.) Various concentrations of sulfuric acid were used to react (A) 15 mM total peroxides with 250 mM potassium iodide or (B) only 250 mM potassium iodide (n = 3). Error bars represent standard deviation.

The addition of sulfuric acid to the reaction between potassium iodide and peroxide greatly increases the rate. As shown in Figure 4A, low concentrations of sulfuric acid do not result in a rapid reaction. A fairly wide range of concentrations centered at ∼3% sulfuric acid provide a window for a stable reaction. However, at very high concentrations of sulfuric acid an increase in iodine production is observed. At these high acid concentrations, the potassium iodide itself can react to generate iodine (Figure 4B). This auto-reaction of potassium iodide is only significant if very low levels of total peroxides are being quantitated and this bias can be mitigated by reading the plate quickly after the addition of potassium iodide. From these results and the stability of the generated iodine in various concentrations of sulfuric acid (see Figure S28), a concentration of 3% sulfuric was determined to be most optimal.

**Figure 5:**
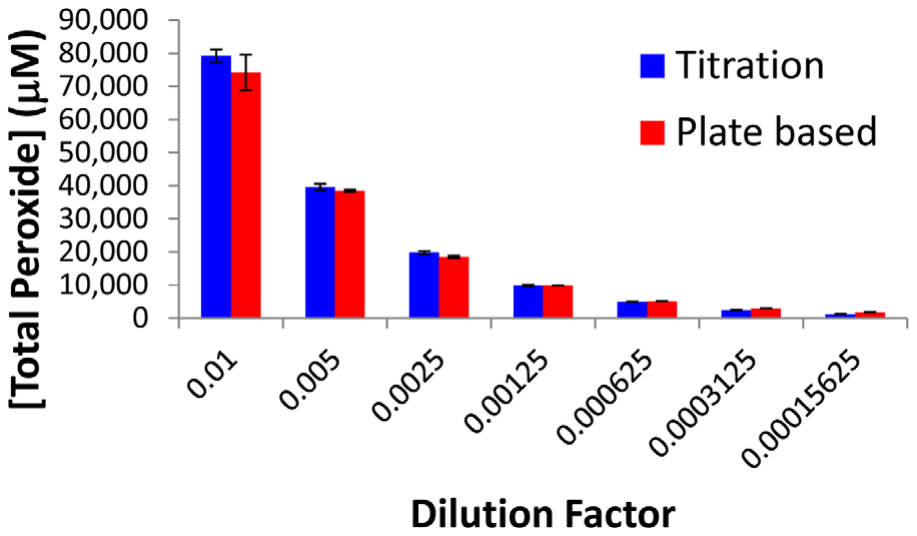
Comparison of total peroxide quantitation. The amount of total peroxide in various dilutions from a stock peracetic acid/H_2_O_2_ solution were determined via titration or the microtiter plate based method (n = 3, error bars represent standard deviation).

To compare the results obtained from the plate based total peroxide detection to those obtained from a titration, several dilutions of a peracetic acid/hydrogen peroxide solution were made and the concentration of total peroxide was determined by both methods. As can be seen in Figure 5, the two methods were in good agreement generally being within the error of one another. However, the values reported are total peroxides. To yield the concentration of peracetic acid alone, the values obtained from the specific hydrogen peroxide method can be subtracted from the total peroxide concentration.

### Peracetic acid – Hydrogen Peroxide Degradation

The formation and degradation of both hydrogen peroxide and peracetic acid can proceed down multiple pathways. The hydrogen peroxide can react with acetic acid to form peracetic acid. The hydrogen peroxide can also breakdown into oxygen and water while the peracetic acid can break down into oxygen and acetic acid or reverse its formation reaction and return to hydrogen peroxide and acetic acid. Due to these multiple pathways, which can occur in a pH dependent manner, the degradation time of these two peroxides together in solution can be difficult to calculate.

**Figure 6:**
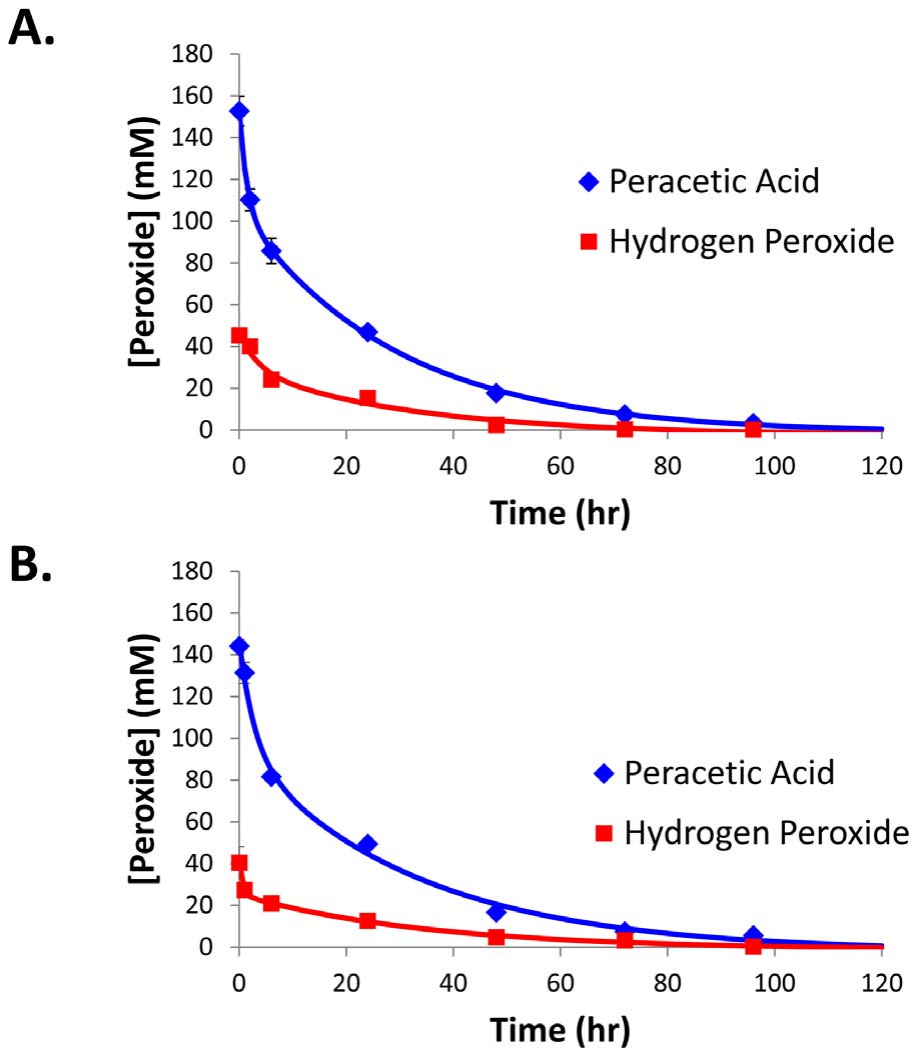
Degradation of peracetic acid and hydrogen peroxide. Three independent peracetic acid/H_2_O_2_ dilutions were created in PBS and initially adjusted to a pH of 7.5. The concentration of peracetic acid and H_2_O_2_ were determined via titration (A.) and the microtiter plate based method. Data represents average of all three dilutions and error bars represent standard deviation.

To show the utility of the microtiter plate based method and to determine the degradation of both peracetic acid and hydrogen peroxide in a PBS solution buffered to physiological pH, the degradation of a peracetic acid/hydrogen peroxide solution was determined by both the microtiter plate and titration methods. As shown in Figure 6, both peracetic acid and hydrogen peroxide rapidly degraded with less than 2% and 0.2% of the starting concentration remaining after 96 hours respectively. Interestingly, the hydrogen peroxide degraded faster than if it were in solution alone. This is mostly likely due to it reacting with the acetic acid in solution to form more peracetic acid which then in turn degrades to acetic acid and oxygen. It should also be noted that the degradation curves from the two different methods yielded very similar results for both peracetic acid and hydrogen peroxide.

## Conclusions

The quantitation of peracetic acid and hydrogen peroxide is of great importance in many industries. However, the availability of rapid quantitation methods that can determine both peroxides in numerous samples was found to be lacking. We were able to adapt the most widely used titration chemistries, the conversion of cerium (IV) sulfate to cerium (III) sulfate and potassium iodide to iodine for use in a two step absorbance microtiter plate method. This new plate based method could be used to rapidly determine the concentration of both hydrogen peroxide and peracetic acid in numerous samples and was found to closely agree with the values obtained during titrations for total peroxides, peracetic acid and hydrogen peroxide. The utility of this method was demonstrated by querying the degradation profiles of hydrogen peroxide and peracetic acid in physiological buffer conditions. In summary, a new, convenient, rapid, absorbance based high-throughput method for the determination of hydrogen peroxide and peracetic acid based upon titration chemistries has been developed.

## Supporting Information

Figure S1 **Peracetic acid/hydrogen peroxide absorbance spectra.**
(TIF)

Figure S2 **Iodine absorbance spectra.**
(TIF)

Figure S3 **Potassium iodide absorbance spectra.**
(TIF)

Figure S4 **Cerium (IV) sulfate absorbance spectra.**
(TIF)

Figure S5 **Cerium (III) sulfate absorbance spectra.**
(TIF)

Figure S6 **Sulfuric acid absorbance spectra.**
(TIF)

Figure S7 **Cerium (IV) sulfate standard absorbance curve at 300** **nm.**
(TIF)

Figure S8 **Cerium (IV) sulfate standard absorbance curve at 325** **nm.**
(TIF)

Figure S9 **Cerium (IV) sulfate standard absorbance curve at 350** **nm.**
(TIF)

Figure S10 **Cerium (IV) sulfate standard absorbance curve at 375** **nm.**
(TIF)

Figure S11 **Cerium (IV) sulfate standard absorbance curve at 400** **nm.**
(TIF)

Figure S12 **Cerium (IV) sulfate standard absorbance curve at 425** **nm.**
(TIF)

Figure S13 **Cerium (IV) sulfate standard absorbance curve at 450** **nm.**
(TIF)

Figure S14 **Cerium (IV) sulfate standard absorbance curve at 475** **nm.**
(TIF)

Figure S15 **Cerium (IV) sulfate standard absorbance curve at 500** **nm.**
(TIF)

Figure S16 **Stability of cerium (IV) sulfate after reaction with peroxide.**
(TIF)

Figure S17 **Iodine standard absorbance curve at 350** **nm.**
(TIF)

Figure S18 **Iodine standard absorbance curve at 375** **nm.**
(TIF)

Figure S19 **Iodine standard absorbance curve at 400** **nm.**
(TIF)

Figure S20 **Iodine standard absorbance curve at 425** **nm.**
(TIF)

Figure S21 **Iodine standard absorbance curve at 450** **nm.**
(TIF)

Figure S22 **Iodine standard absorbance curve at 475** **nm.**
(TIF)

Figure S23 **Iodine standard absorbance curve at 500** **nm.**
(TIF)

Figure S24 **Iodine standard absorbance curve at 525** **nm.**
(TIF)

Figure S25 **Iodine standard absorbance curve at 550nm.**
(TIF)

Figure S26 **Iodine standard absorbance curve at 575** **nm.**
(TIF)

Figure S27 **Iodine standard absorbance curve at 600** **nm.**
(TIF)

Figure S28 **Stability of iodine generated from peroxide – potassium iodide reaction in various sulfuric acid concentrations.**
(TIF)
