# Supplementary figures and images for: A High-Throughput Microtiter Plate Based Method for the Determination of Peracetic Acid and Hydrogen Peroxide
Source: PLoS One. 2013 Nov 18;8(11):e79218. doi: 10.1371/journal.pone.0079218 (PMC3832485; doi:10.1371/journal.pone.0079218)

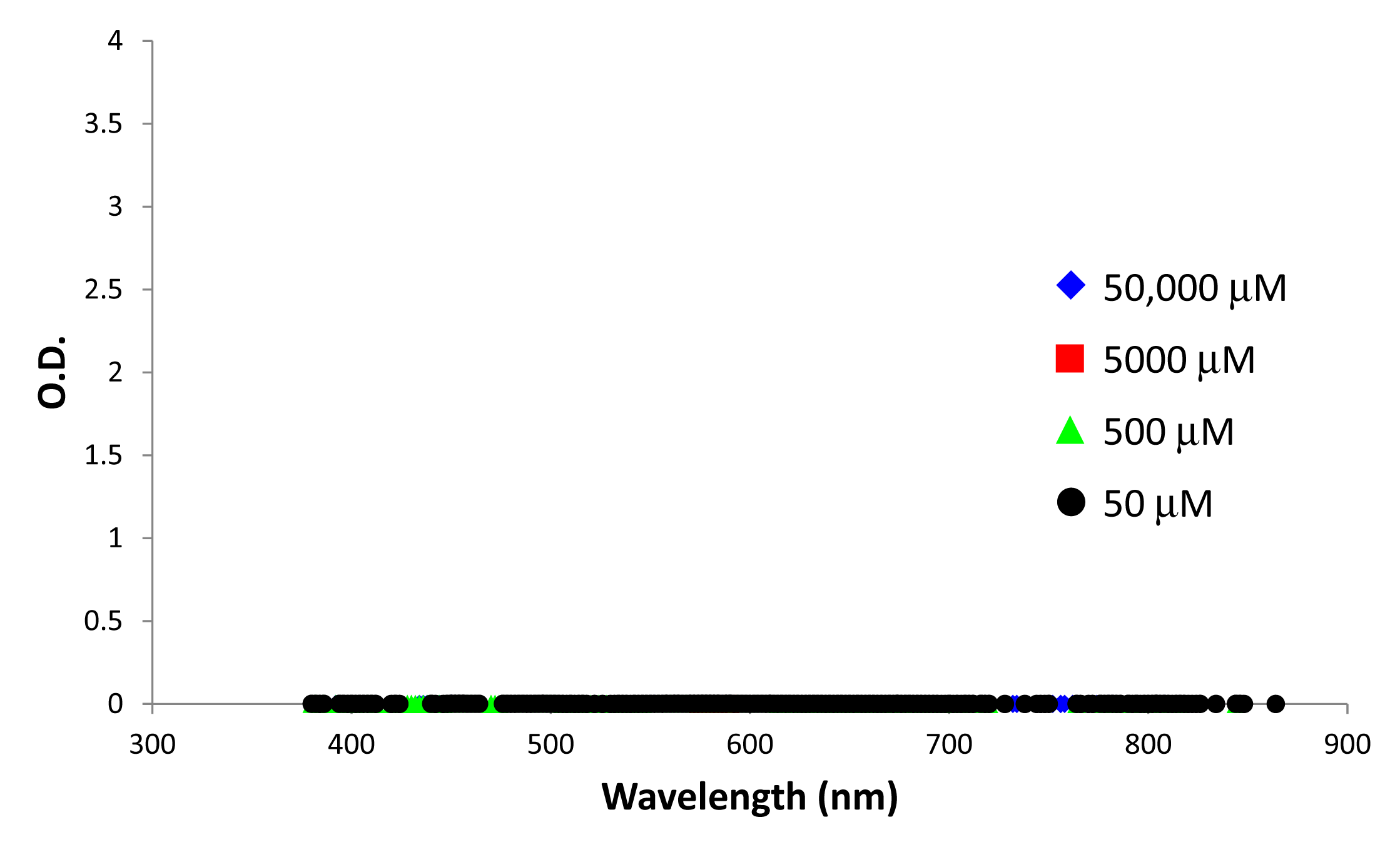

Supplement: Figure S1 — Peracetic acid/hydrogen peroxide absorbance spectra. (TIF) [file pone.0079218.s001.tif]

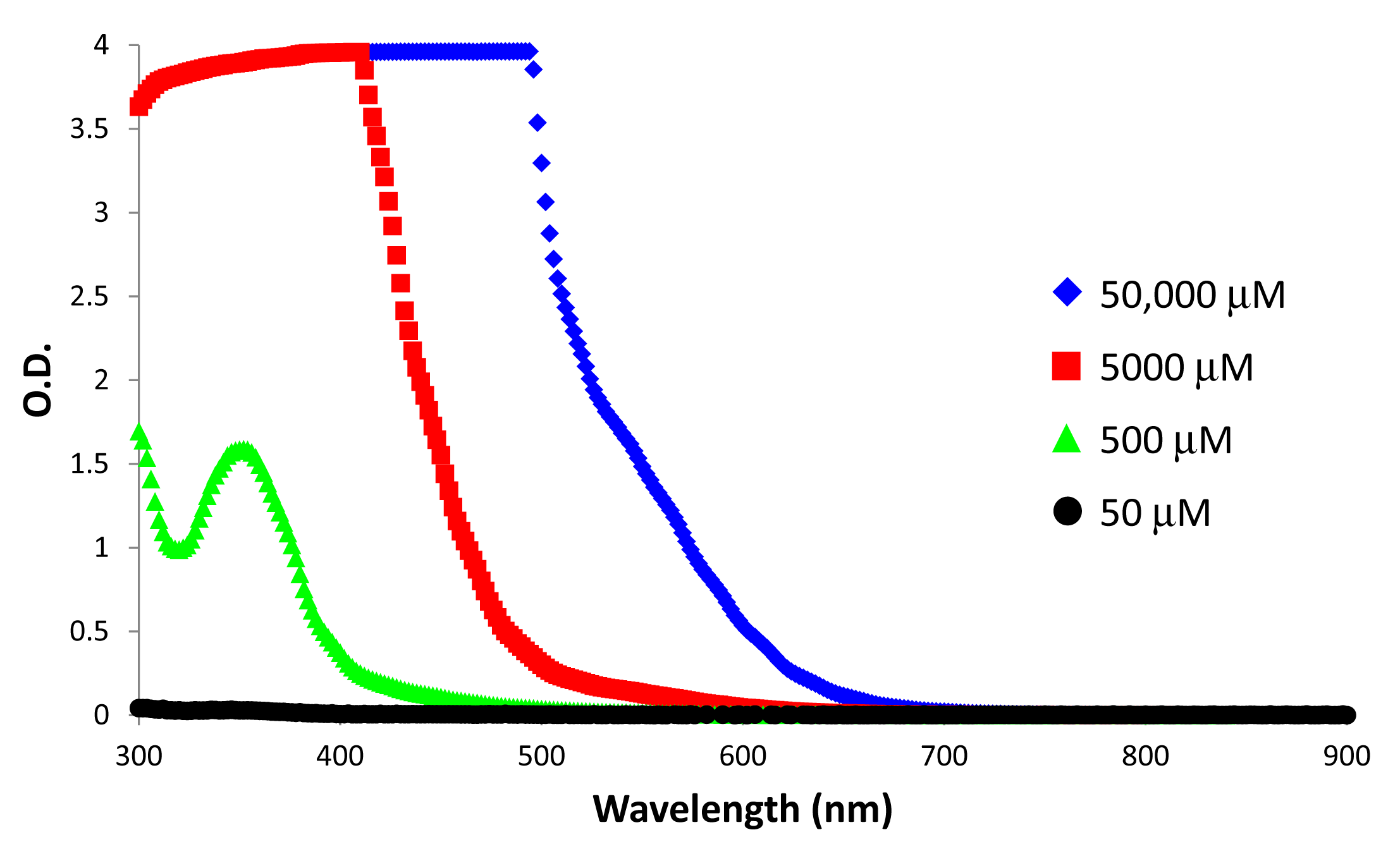

Supplement: Figure S2 — Iodine absorbance spectra. (TIF) [file pone.0079218.s002.tif]

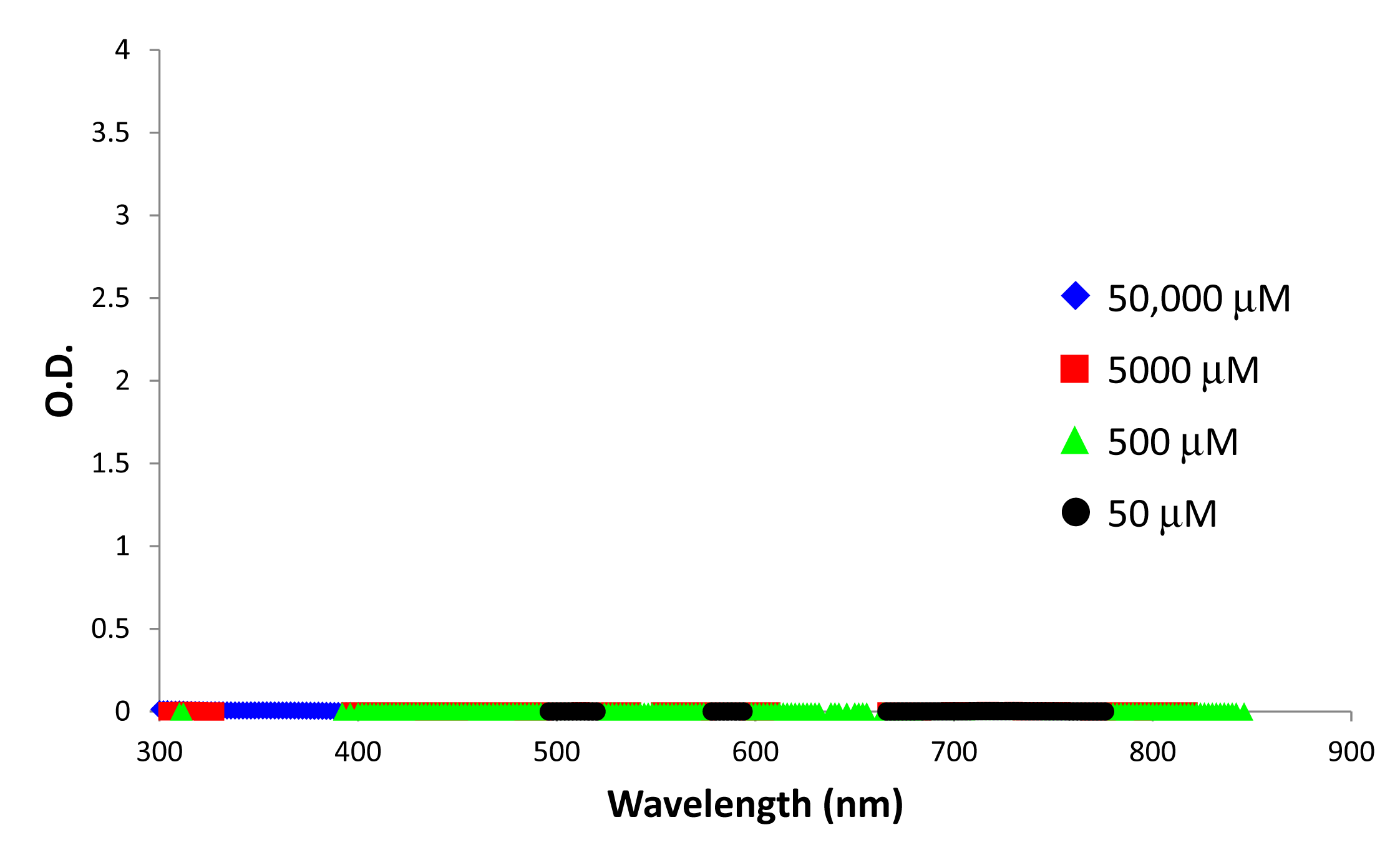

Supplement: Figure S3 — Potassium iodide absorbance spectra. (TIF) [file pone.0079218.s003.tif]

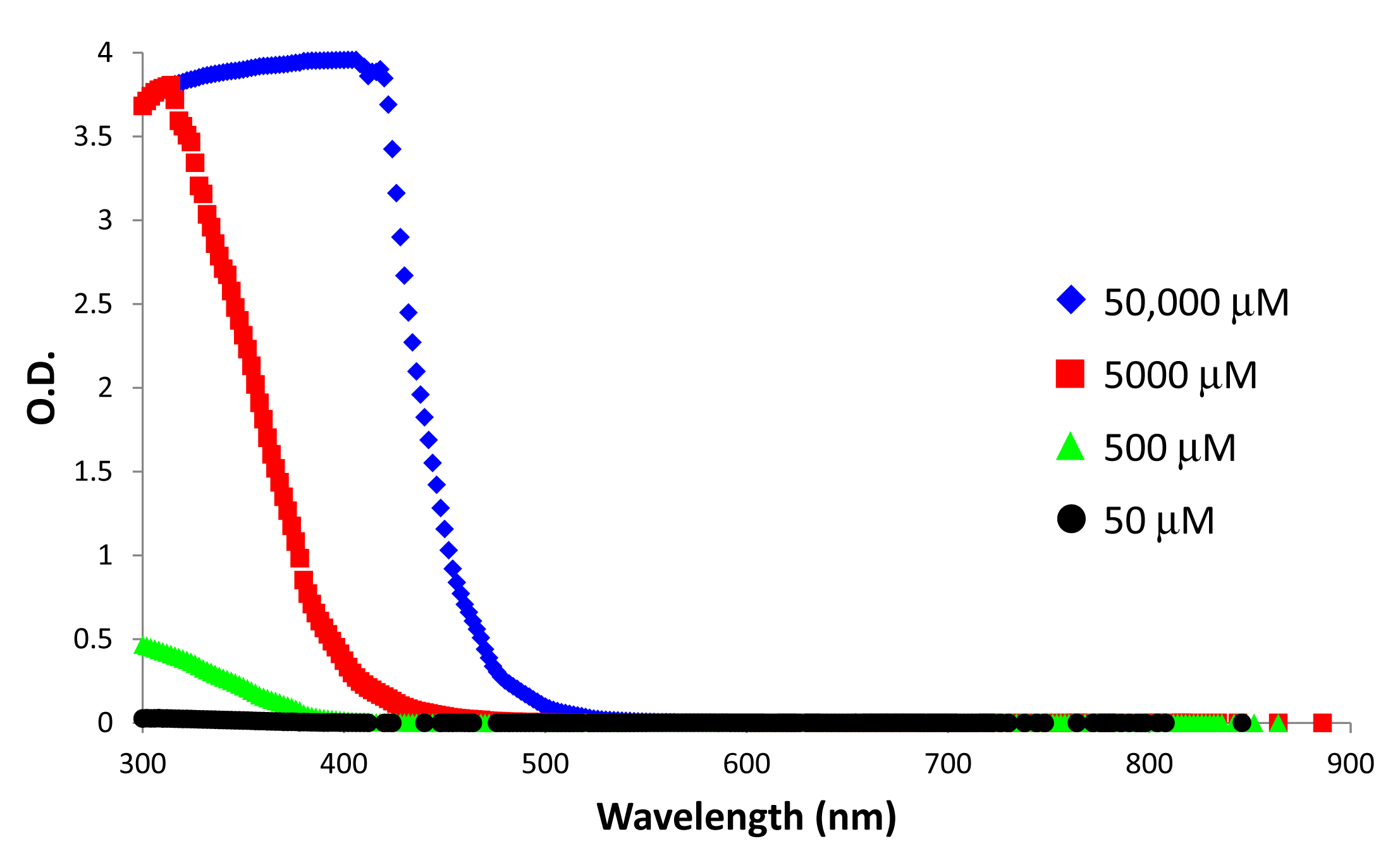

Supplement: Figure S4 — Cerium (IV) sulfate absorbance spectra. (TIF) [file pone.0079218.s004.tif]

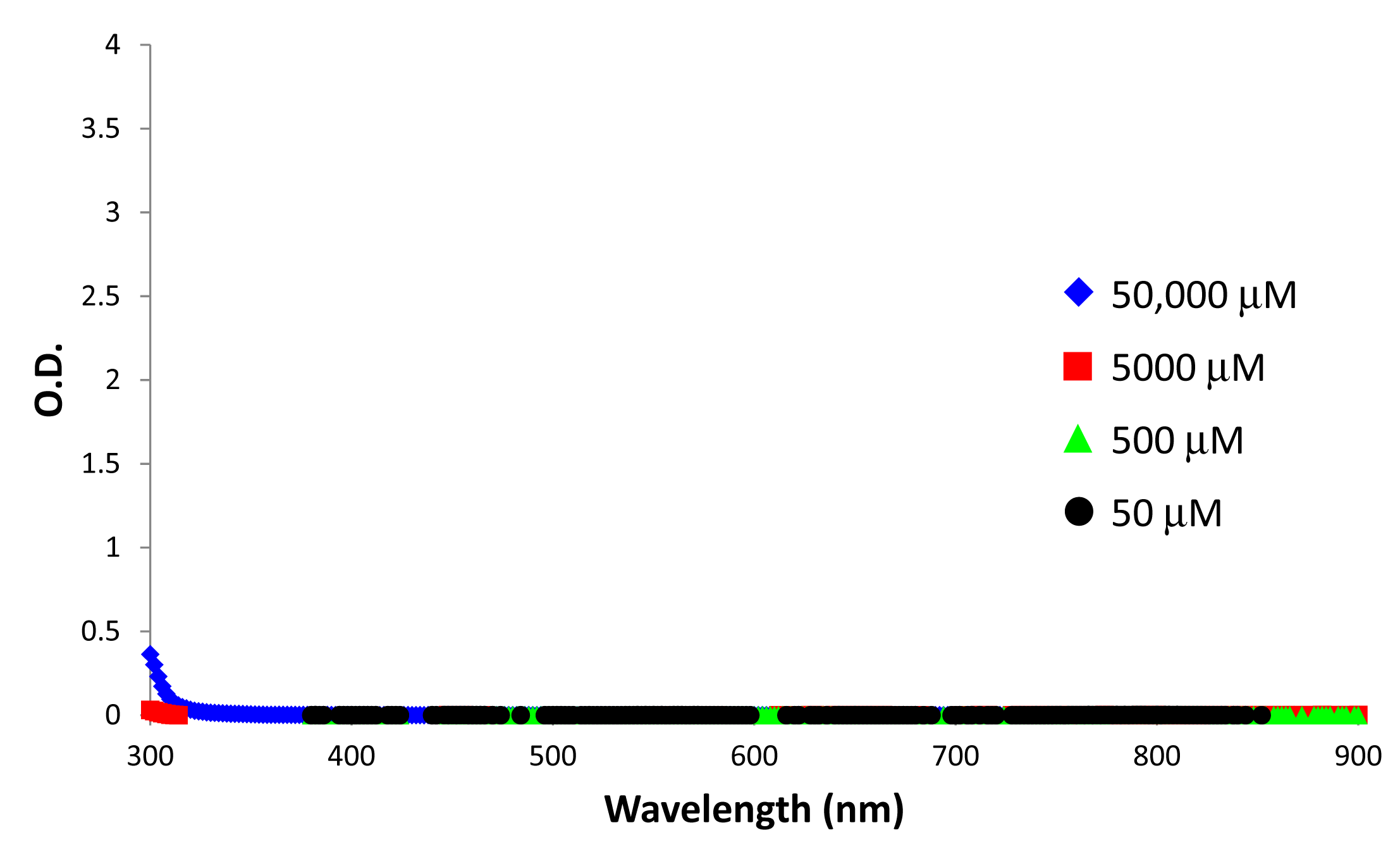

Supplement: Figure S5 — Cerium (III) sulfate absorbance spectra. (TIF) [file pone.0079218.s005.tif]

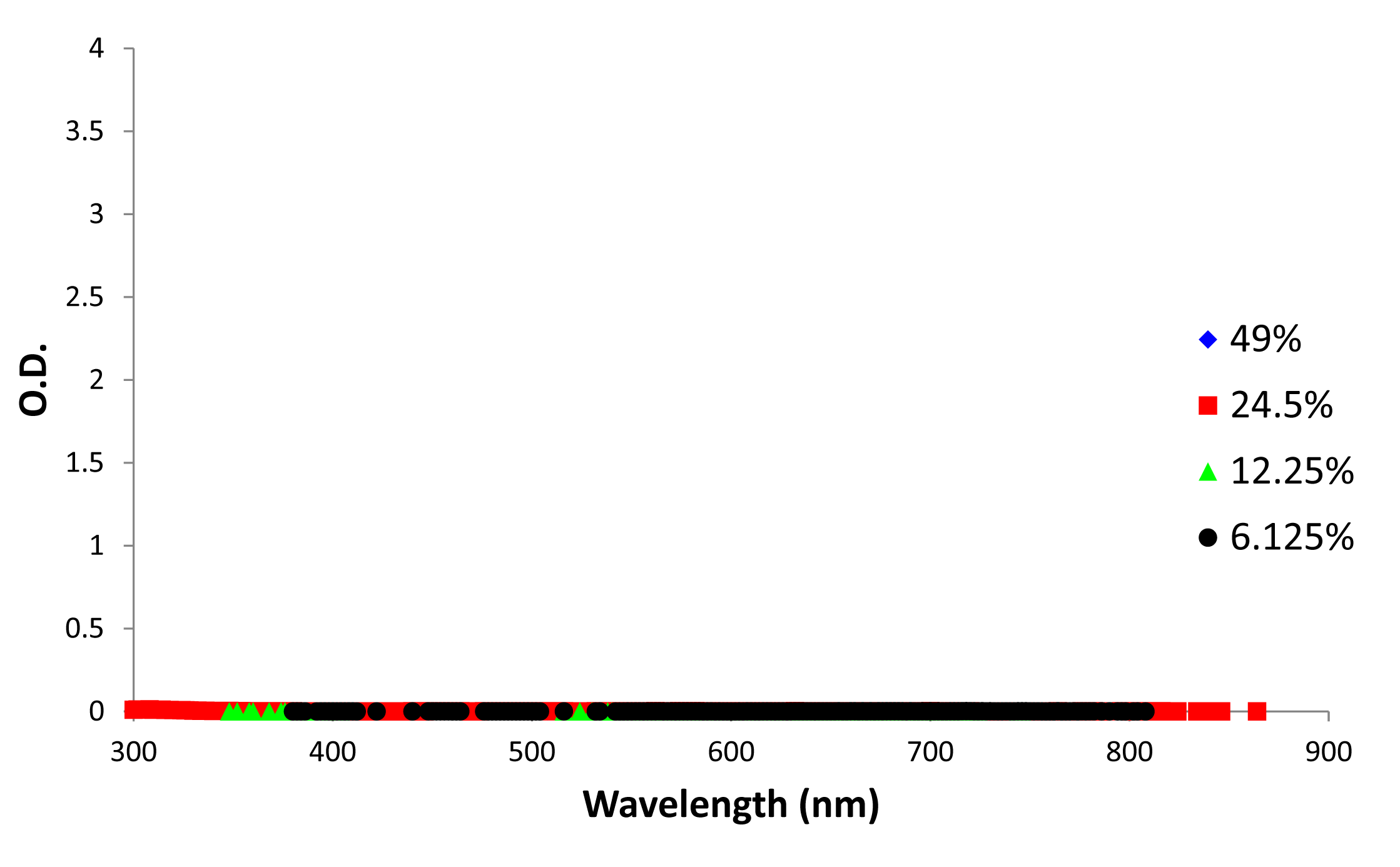

Supplement: Figure S6 — Sulfuric acid absorbance spectra. (TIF) [file pone.0079218.s006.tif]

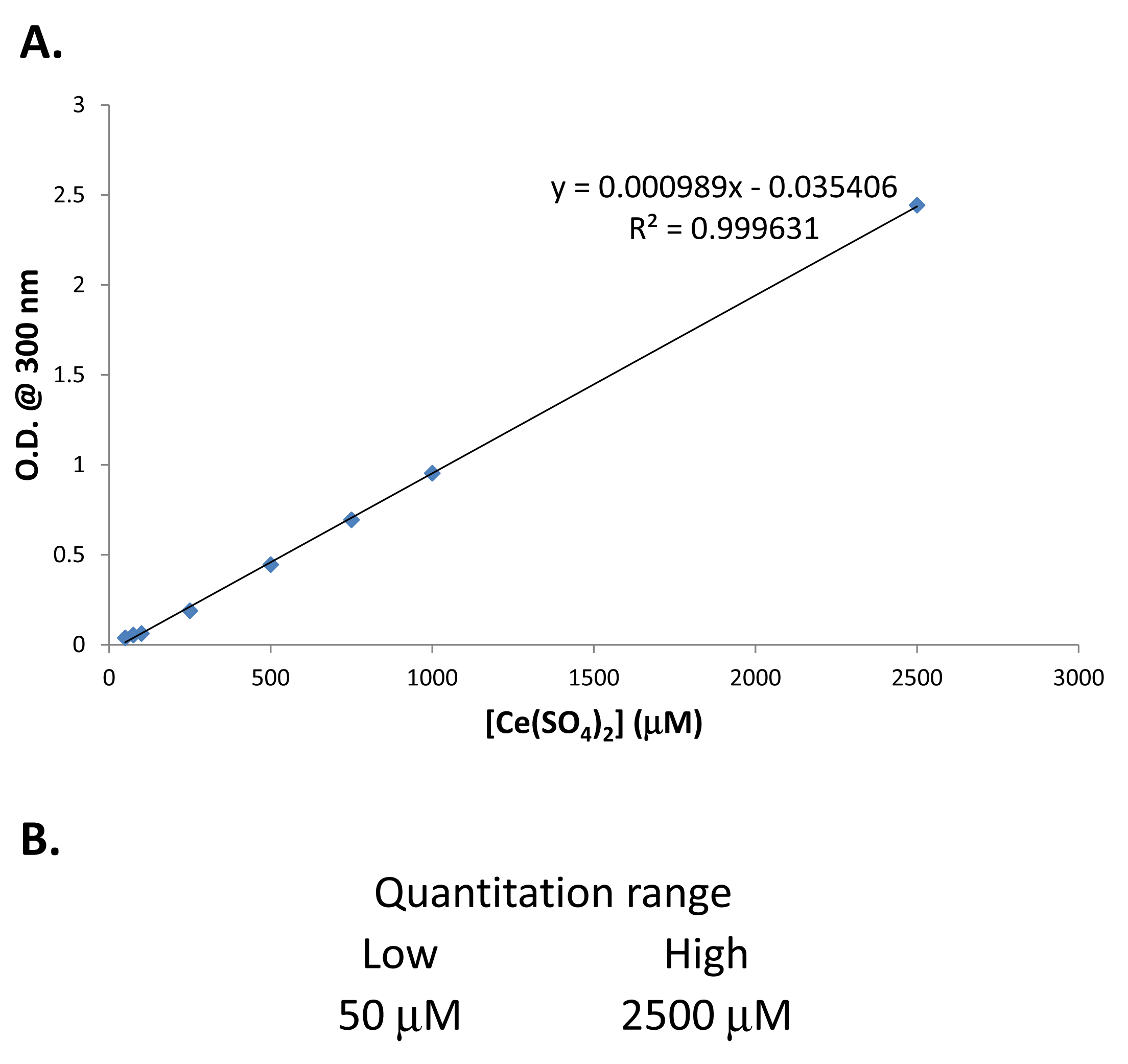

Supplement: Figure S7 — Cerium (IV) sulfate standard absorbance curve at 300 nm. (TIF) [file pone.0079218.s007.tif]

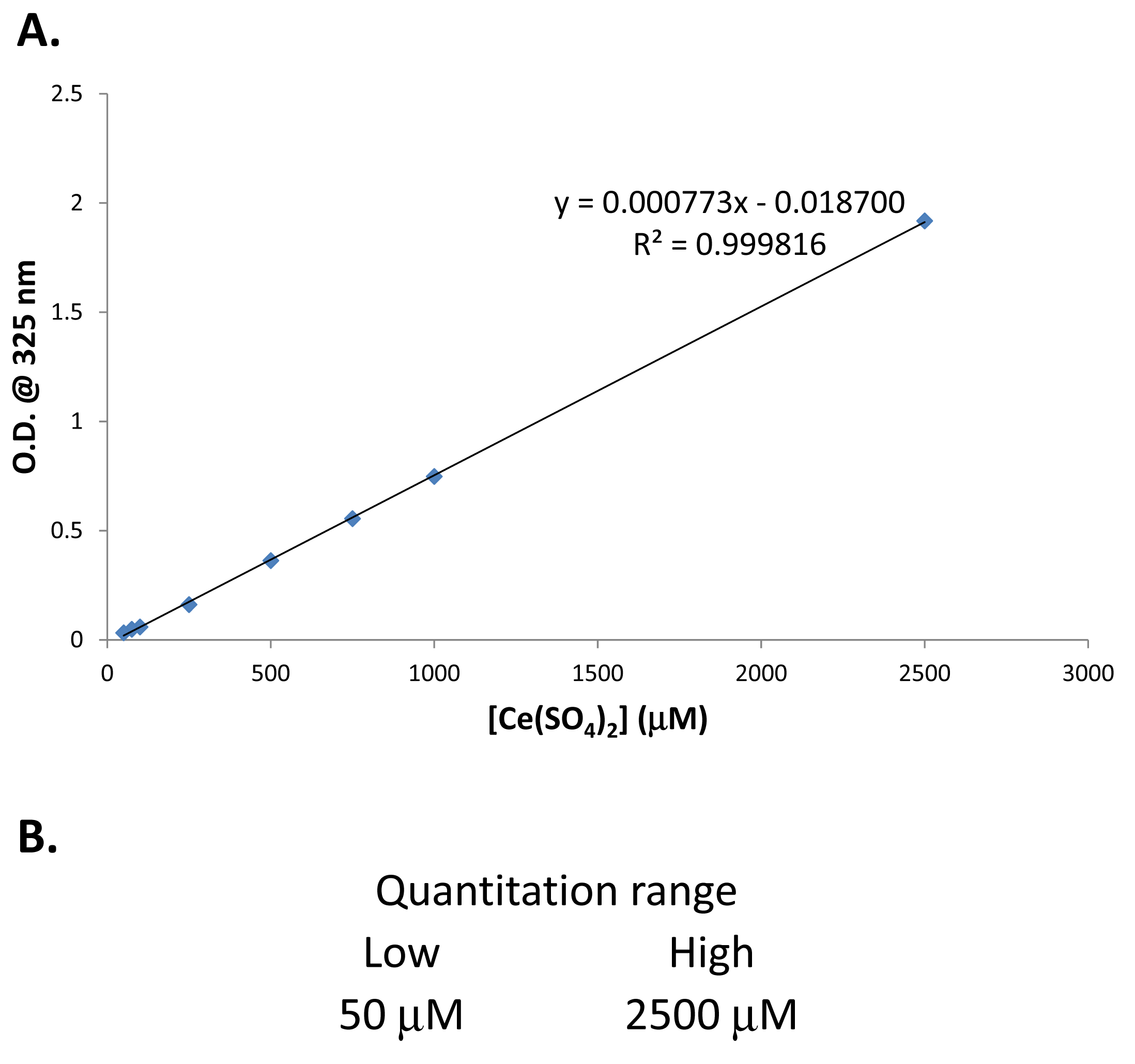

Supplement: Figure S8 — Cerium (IV) sulfate standard absorbance curve at 325 nm. (TIF) [file pone.0079218.s008.tif]

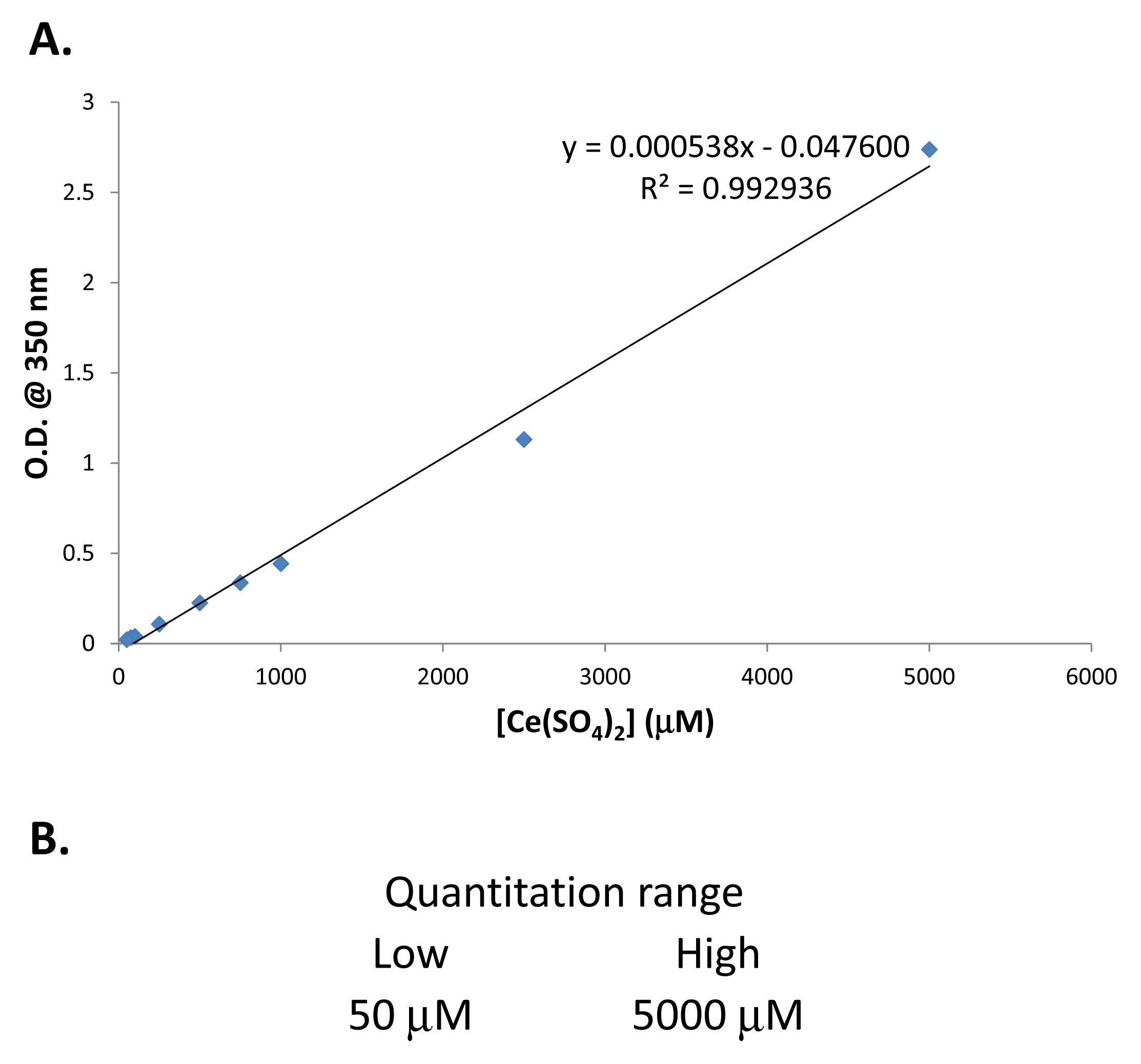

Supplement: Figure S9 — Cerium (IV) sulfate standard absorbance curve at 350 nm. (TIF) [file pone.0079218.s009.tif]

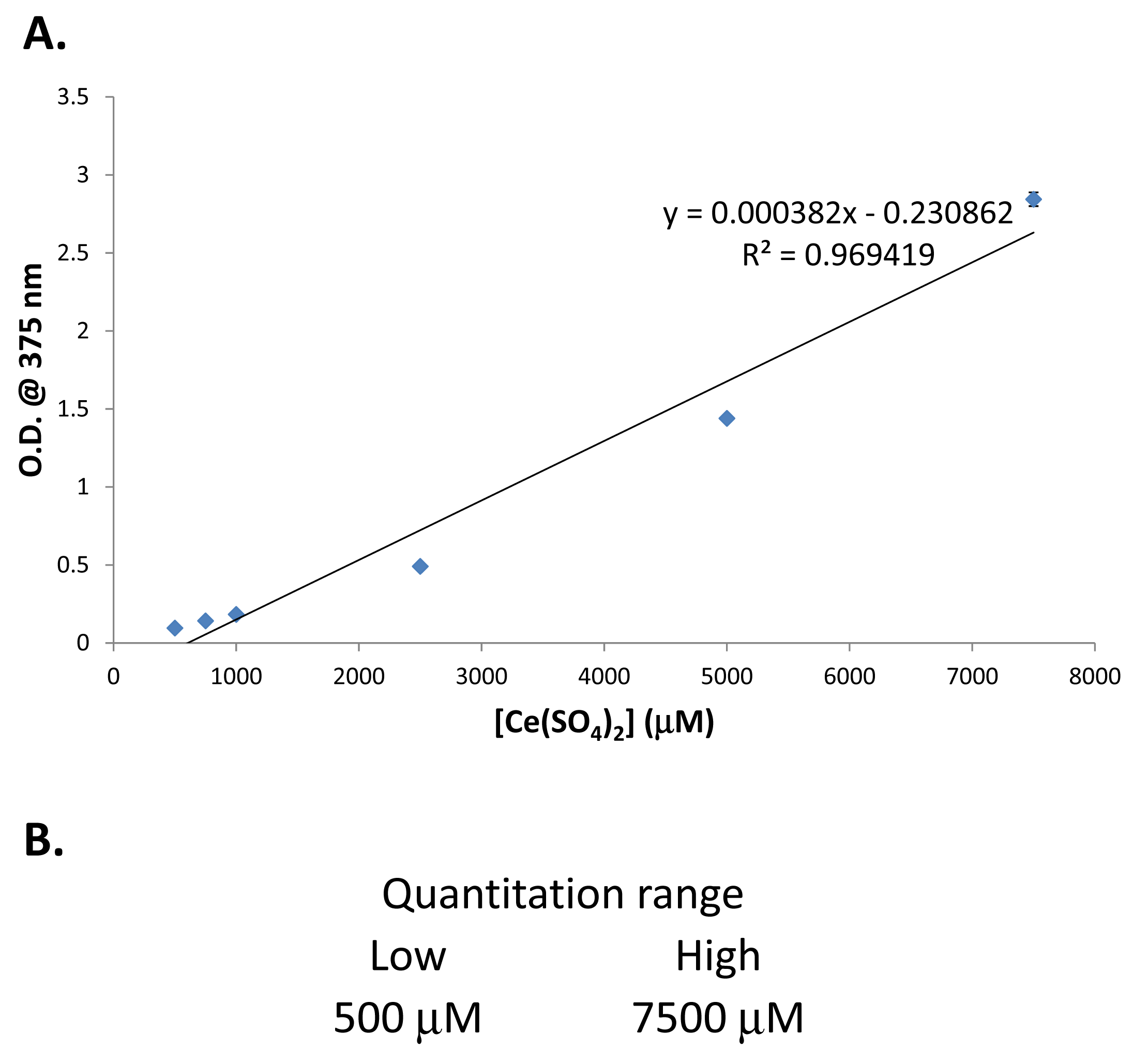

Supplement: Figure S10 — Cerium (IV) sulfate standard absorbance curve at 375 nm. (TIF) [file pone.0079218.s010.tif]

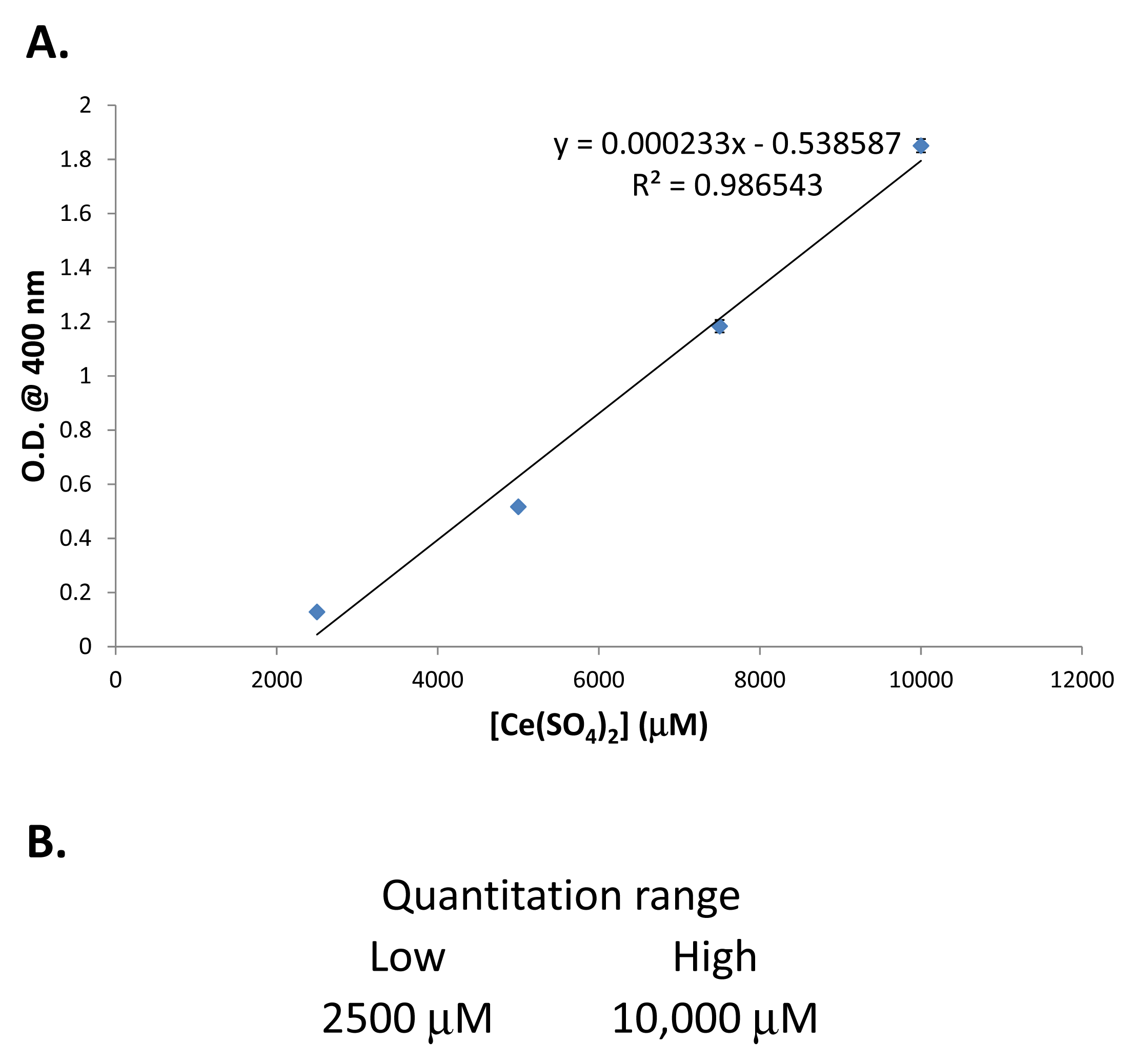

Supplement: Figure S11 — Cerium (IV) sulfate standard absorbance curve at 400 nm. (TIF) [file pone.0079218.s011.tif]

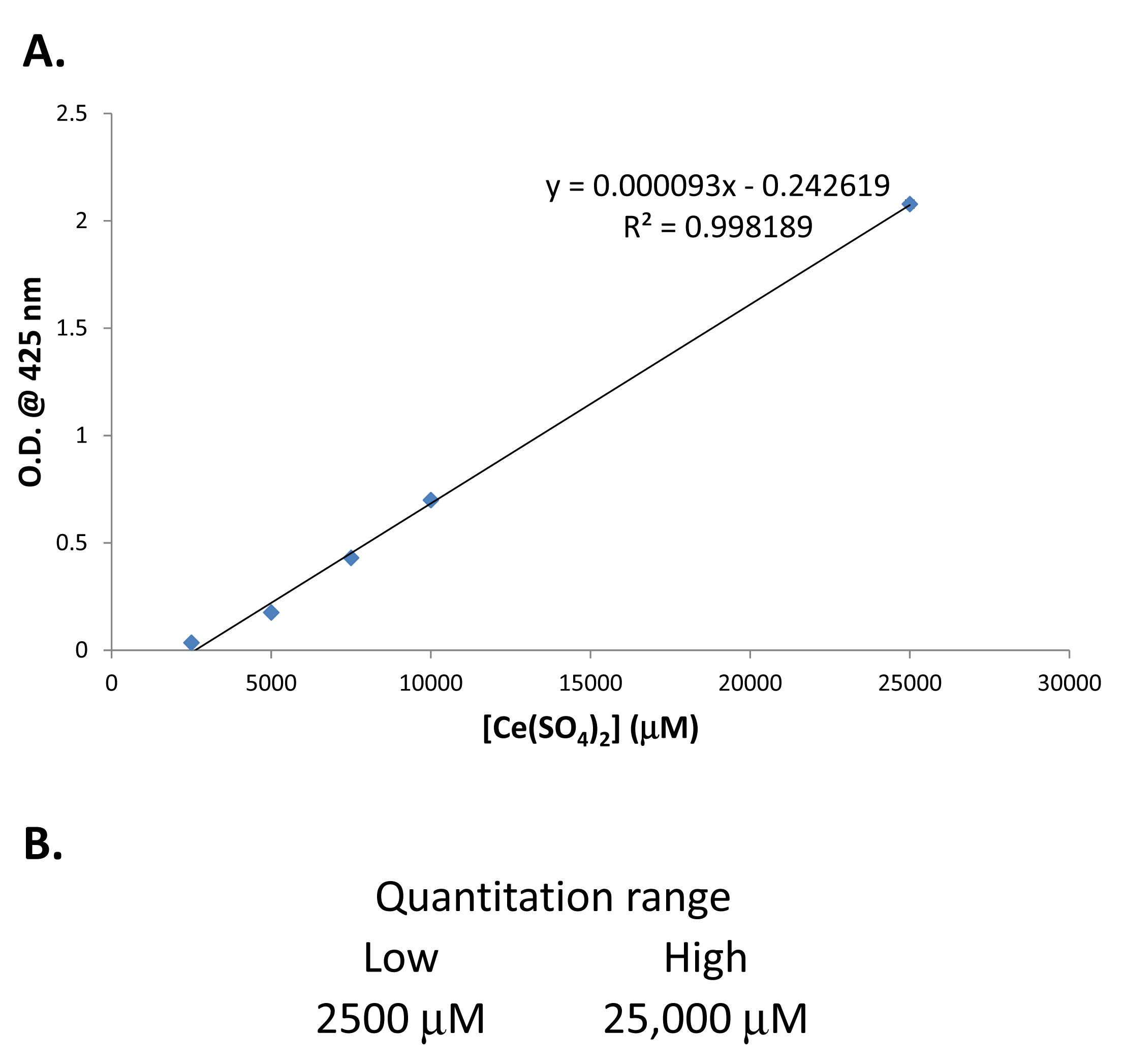

Supplement: Figure S12 — Cerium (IV) sulfate standard absorbance curve at 425 nm. (TIF) [file pone.0079218.s012.tif]

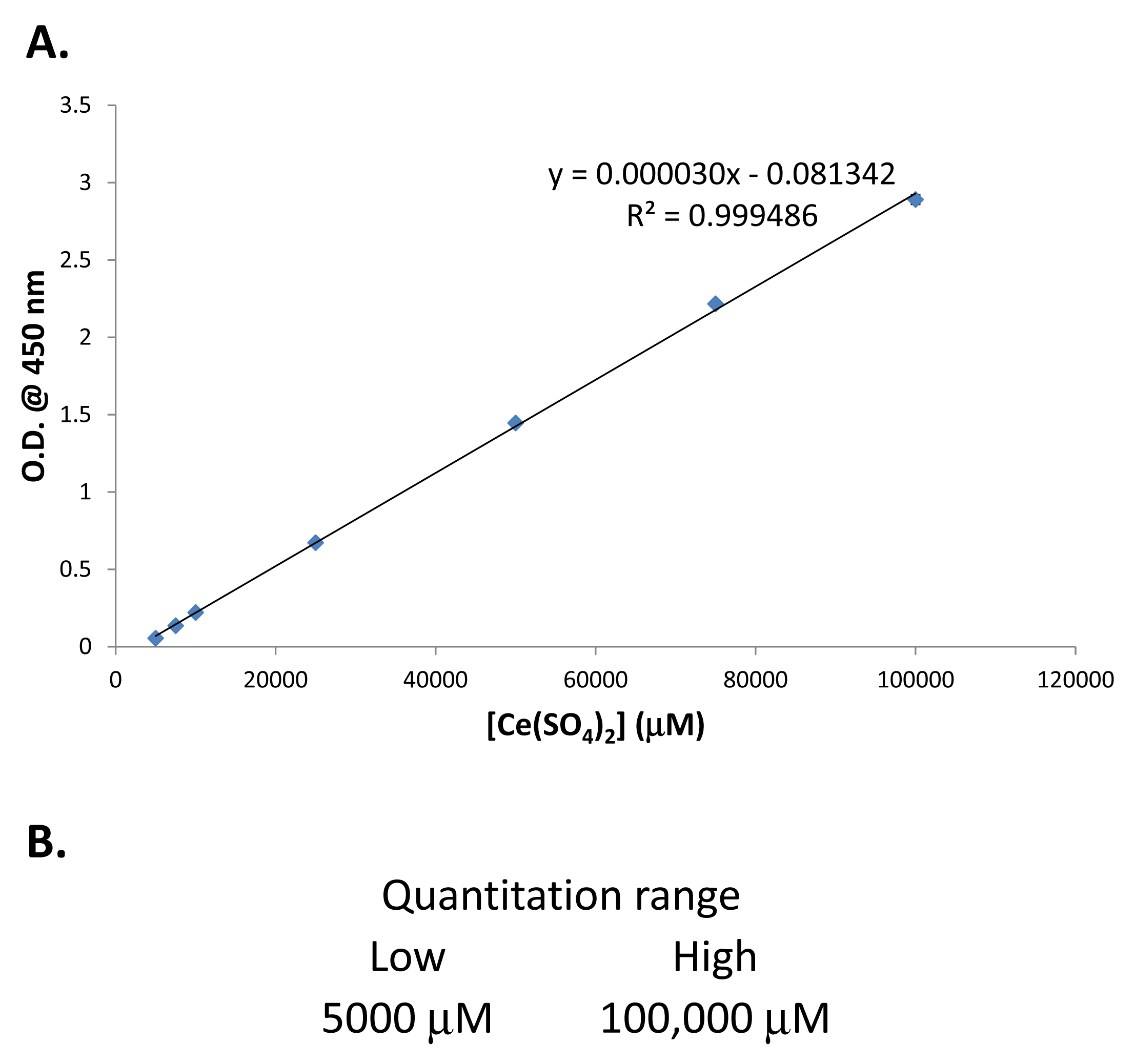

Supplement: Figure S13 — Cerium (IV) sulfate standard absorbance curve at 450 nm. (TIF) [file pone.0079218.s013.tif]

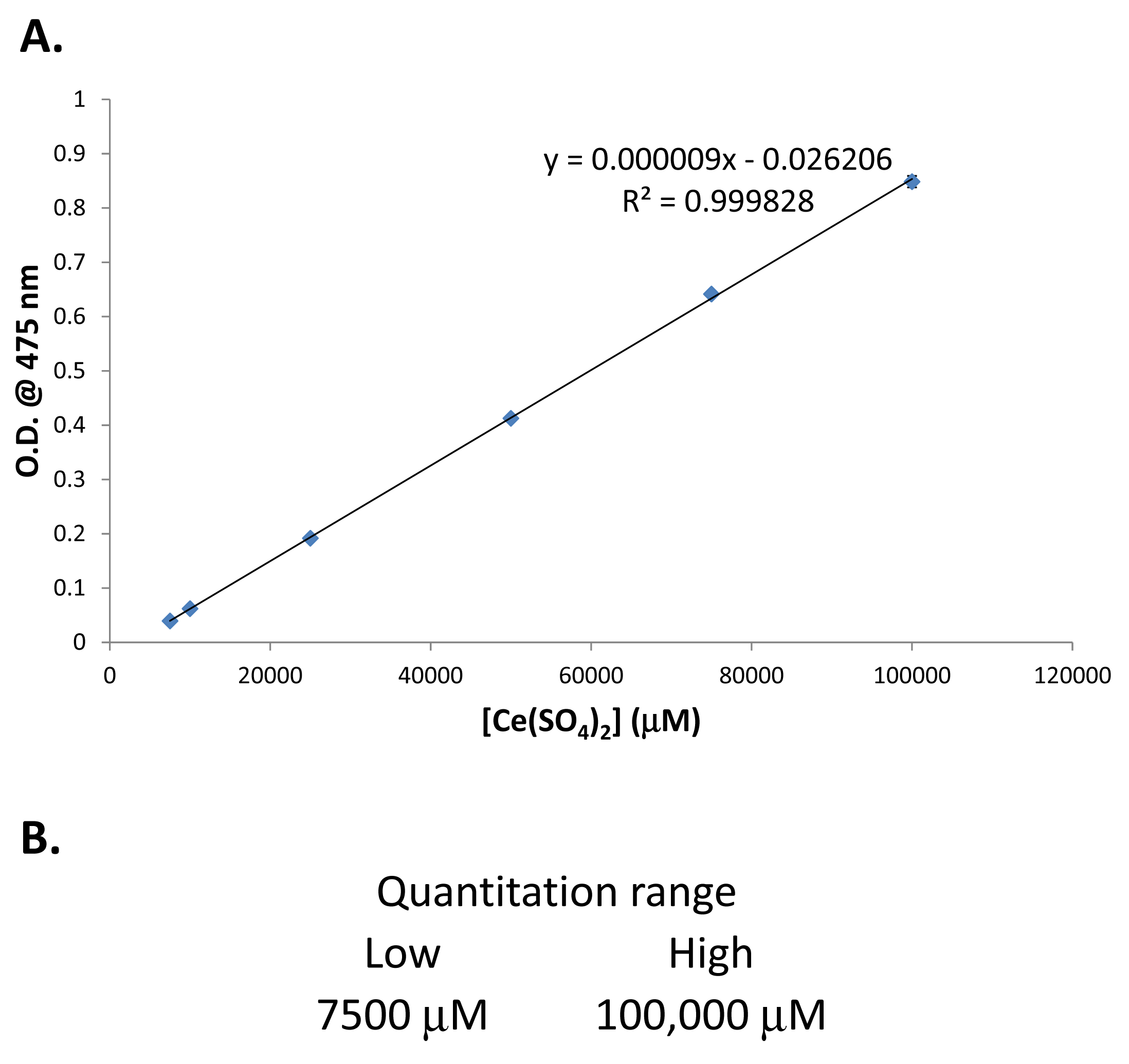

Supplement: Figure S14 — Cerium (IV) sulfate standard absorbance curve at 475 nm. (TIF) [file pone.0079218.s014.tif]

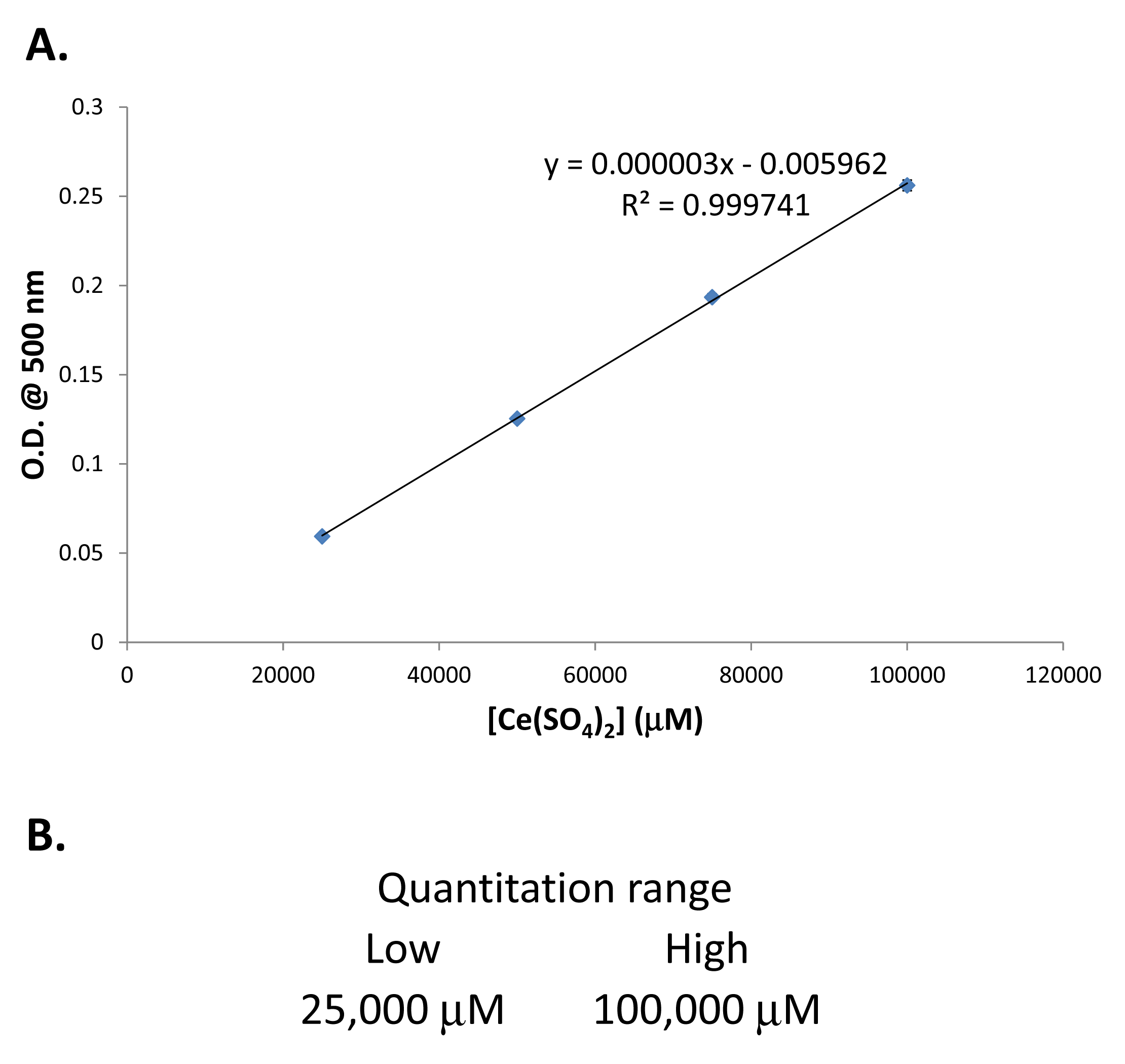

Supplement: Figure S15 — Cerium (IV) sulfate standard absorbance curve at 500 nm. (TIF) [file pone.0079218.s015.tif]

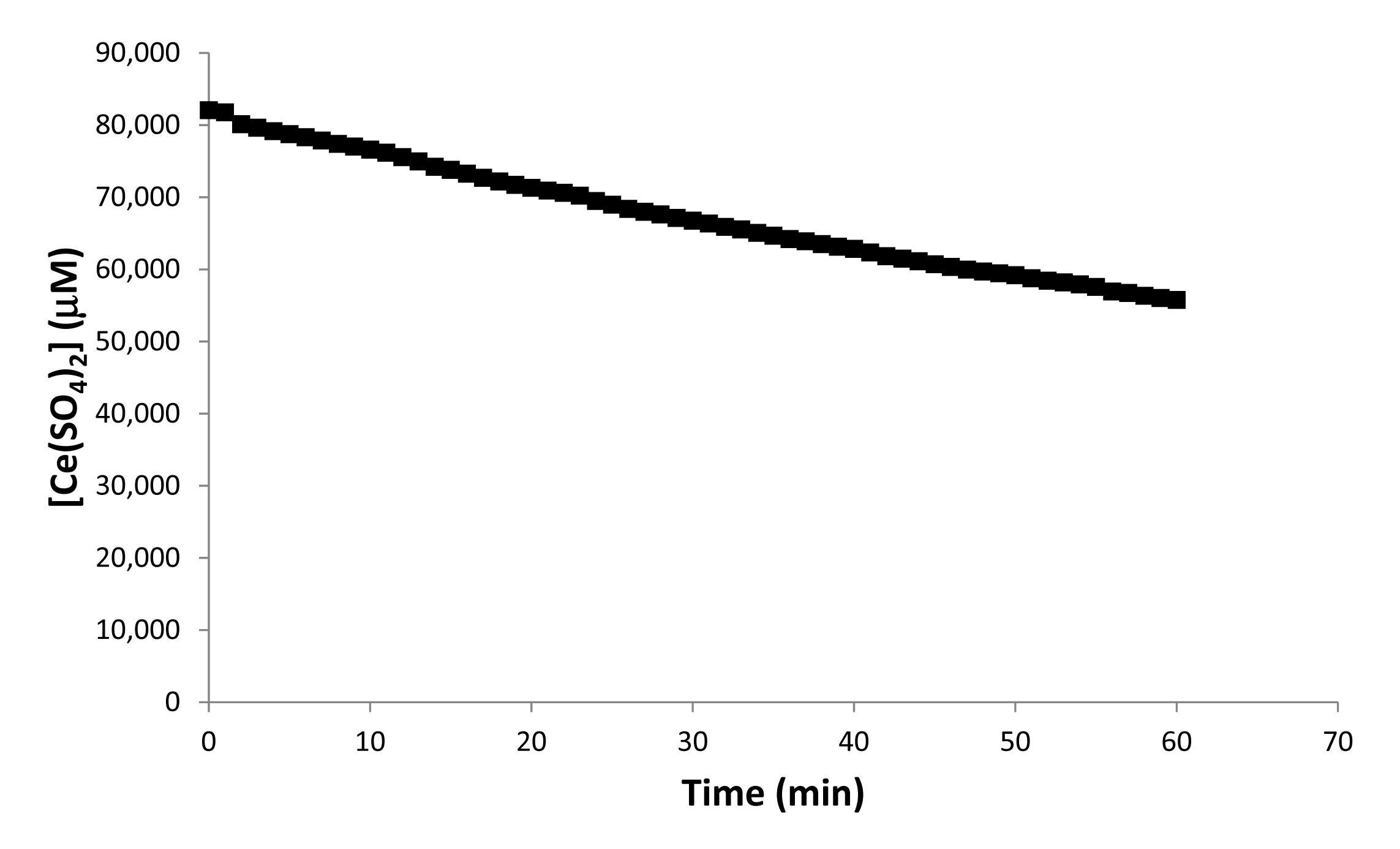

Supplement: Figure S16 — Stability of cerium (IV) sulfate after reaction with peroxide. (TIF) [file pone.0079218.s016.tif]

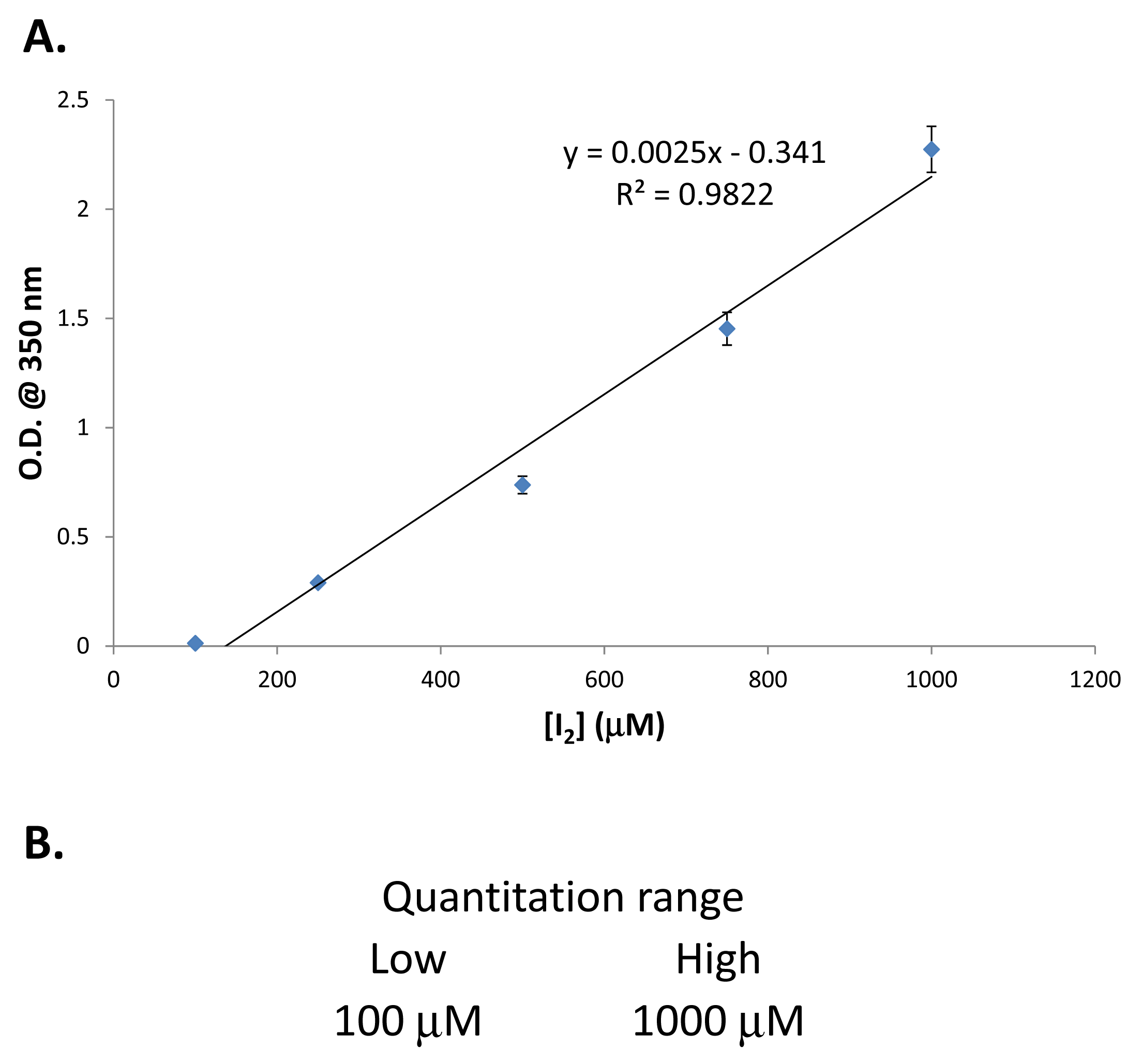

Supplement: Figure S17 — Iodine standard absorbance curve at 350 nm. (TIF) [file pone.0079218.s017.tif]

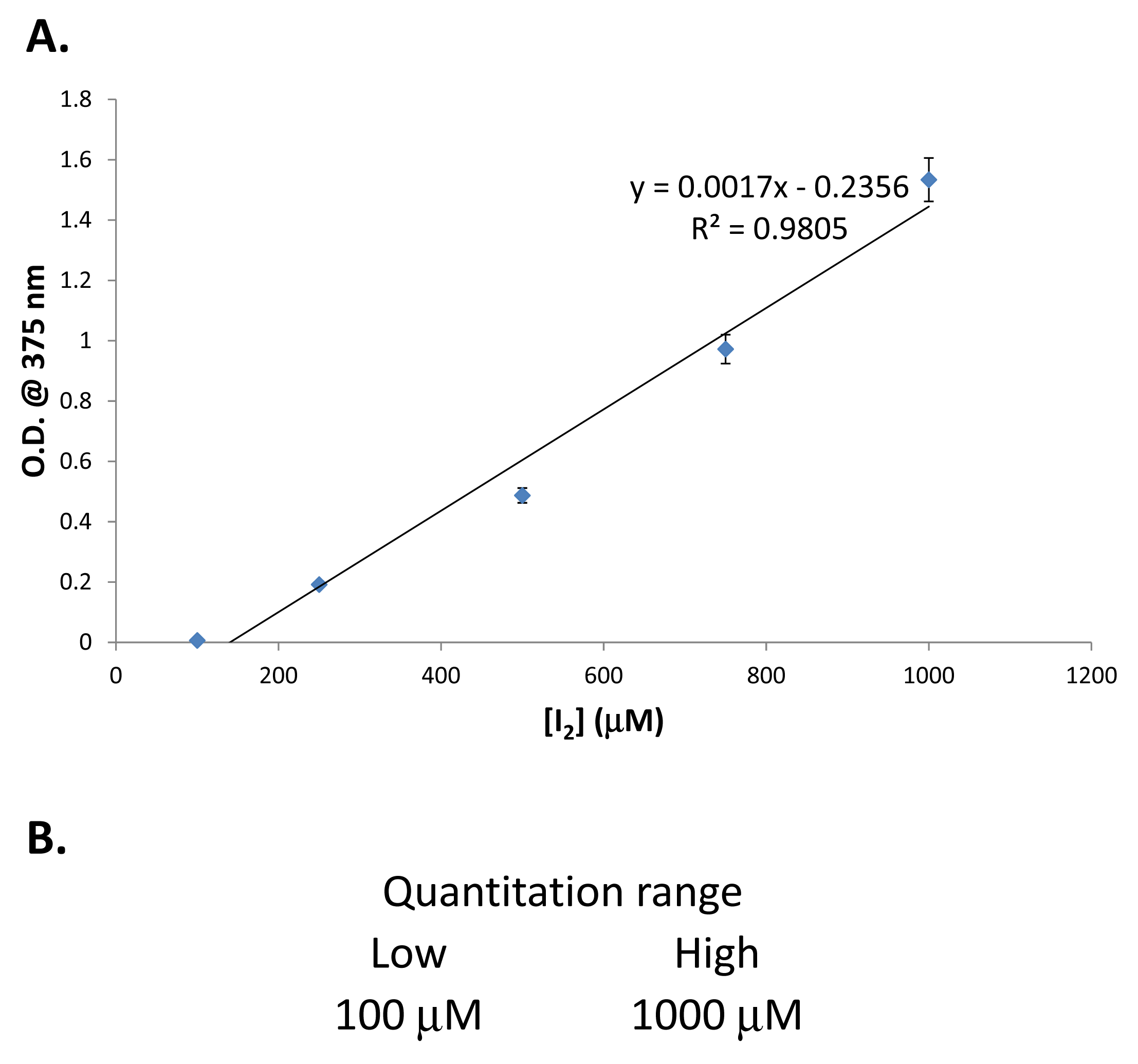

Supplement: Figure S18 — Iodine standard absorbance curve at 375 nm. (TIF) [file pone.0079218.s018.tif]

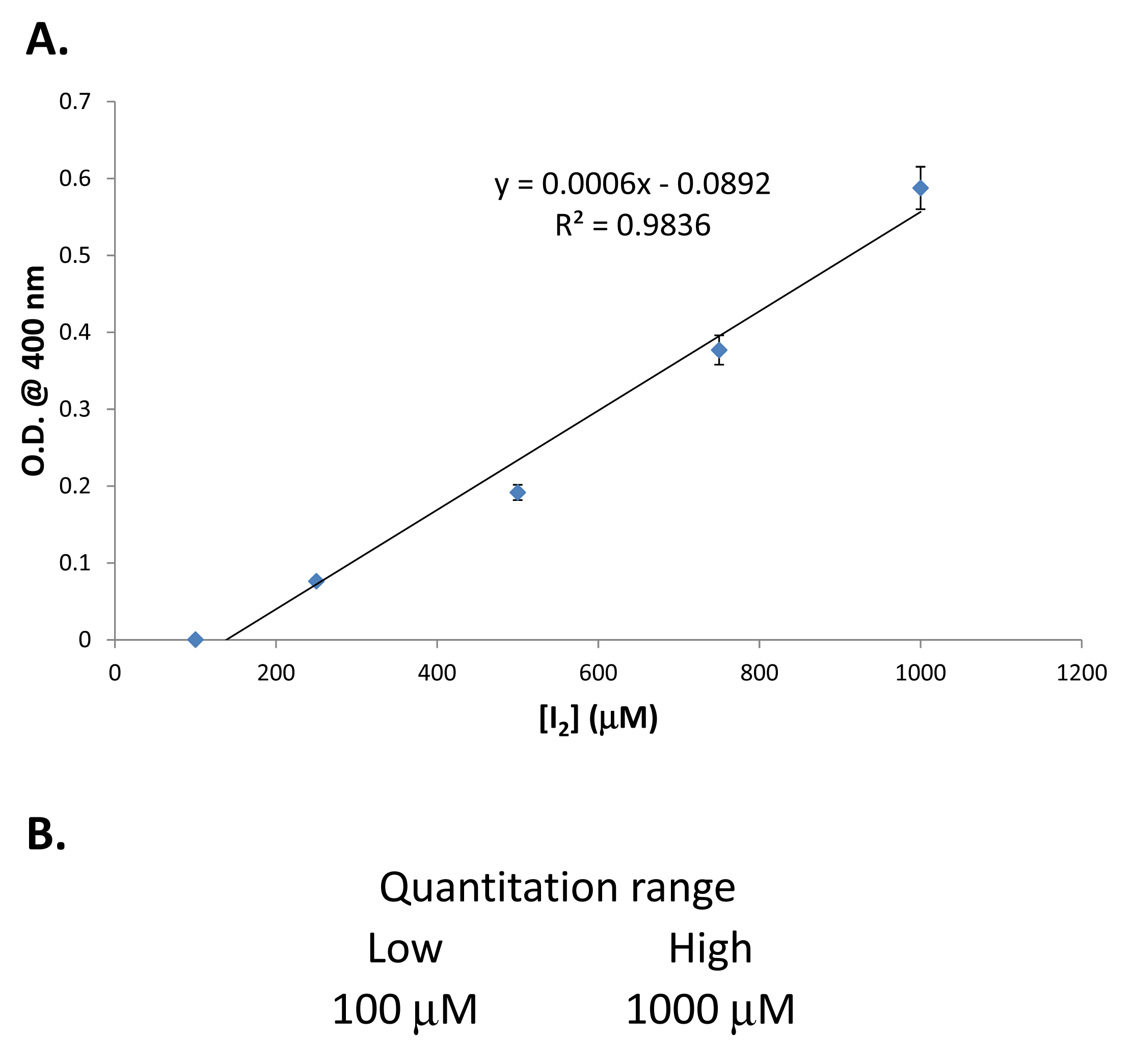

Supplement: Figure S19 — Iodine standard absorbance curve at 400 nm. (TIF) [file pone.0079218.s019.tif]

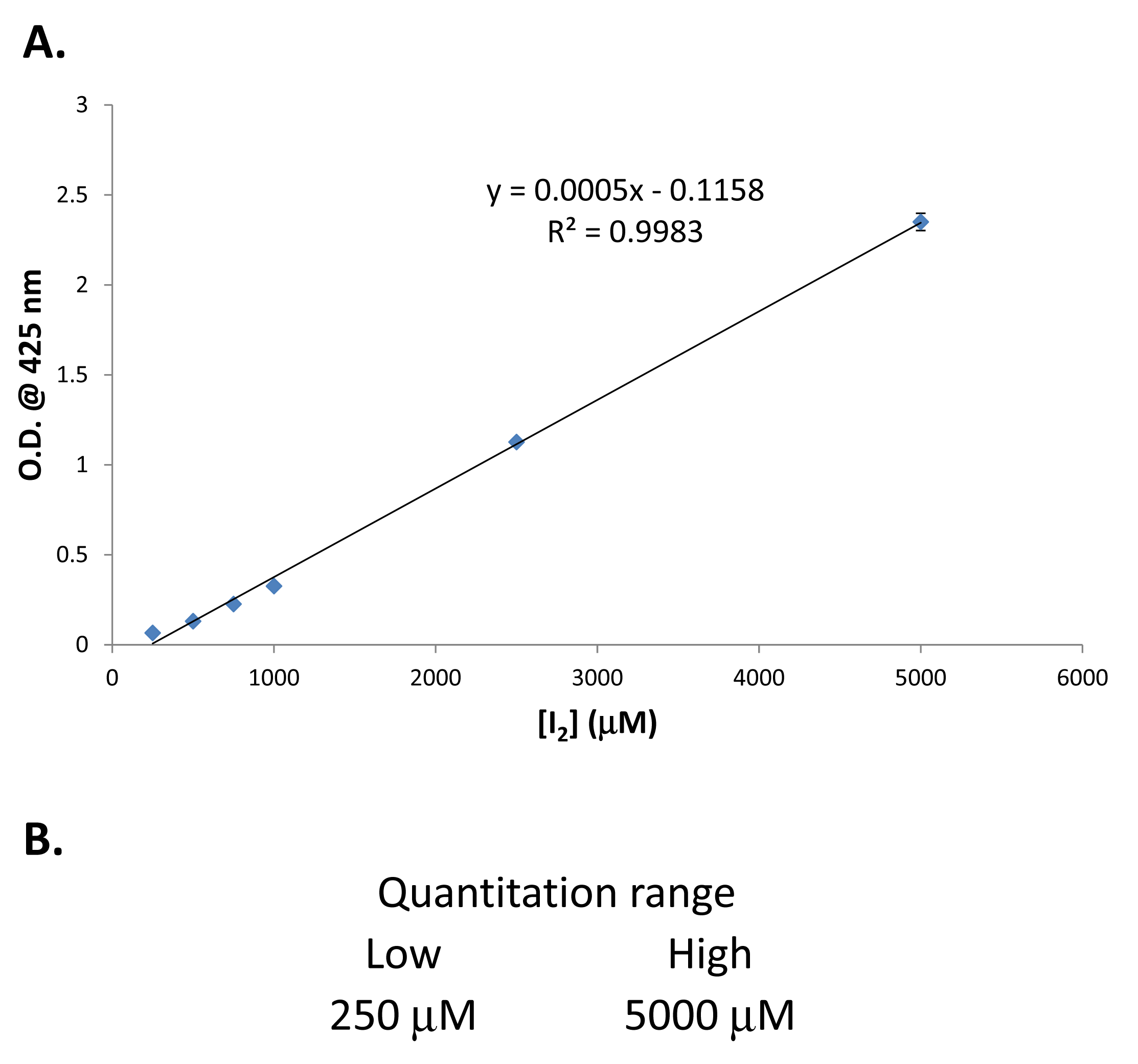

Supplement: Figure S20 — Iodine standard absorbance curve at 425 nm. (TIF) [file pone.0079218.s020.tif]

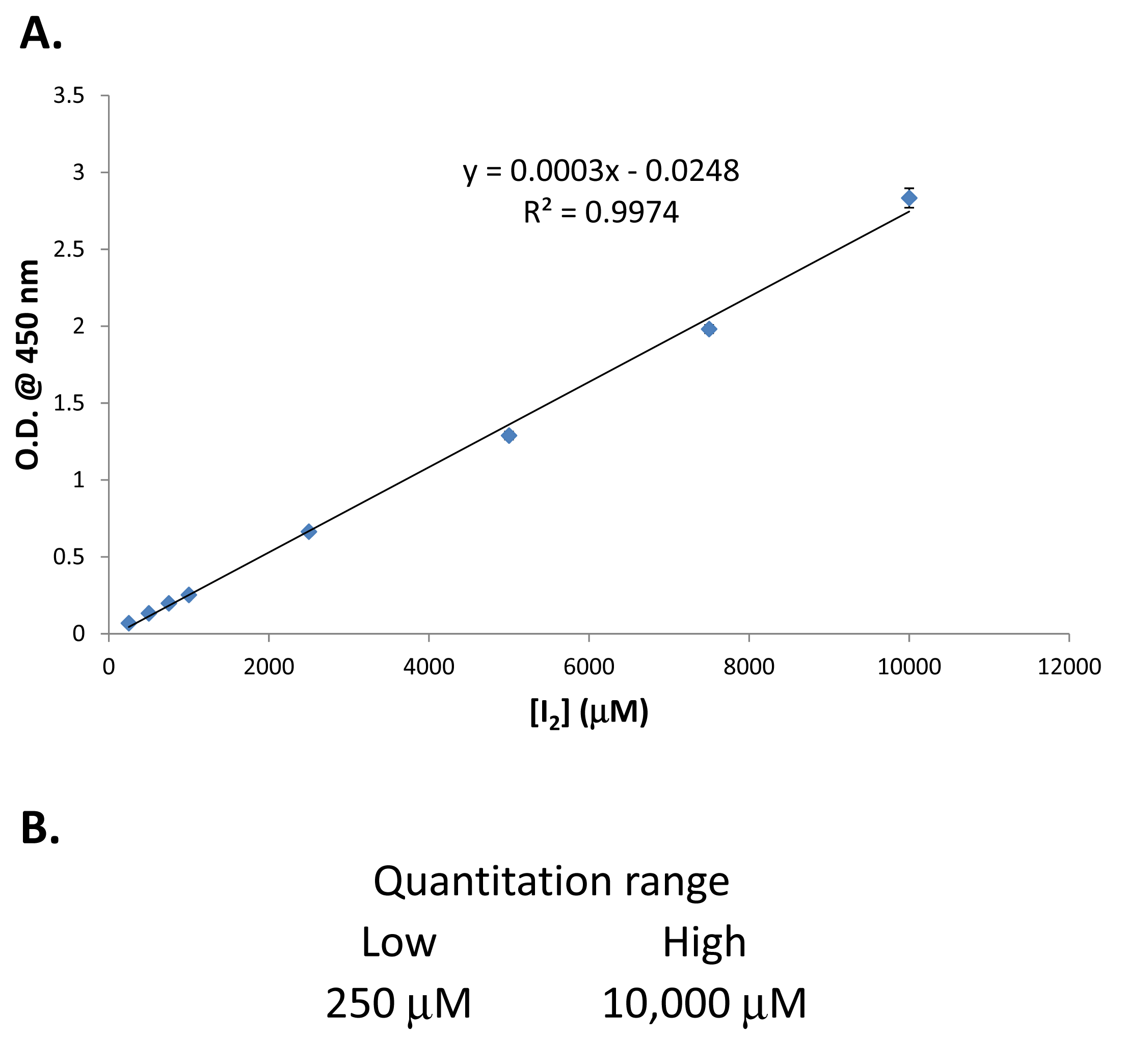

Supplement: Figure S21 — Iodine standard absorbance curve at 450 nm. (TIF) [file pone.0079218.s021.tif]

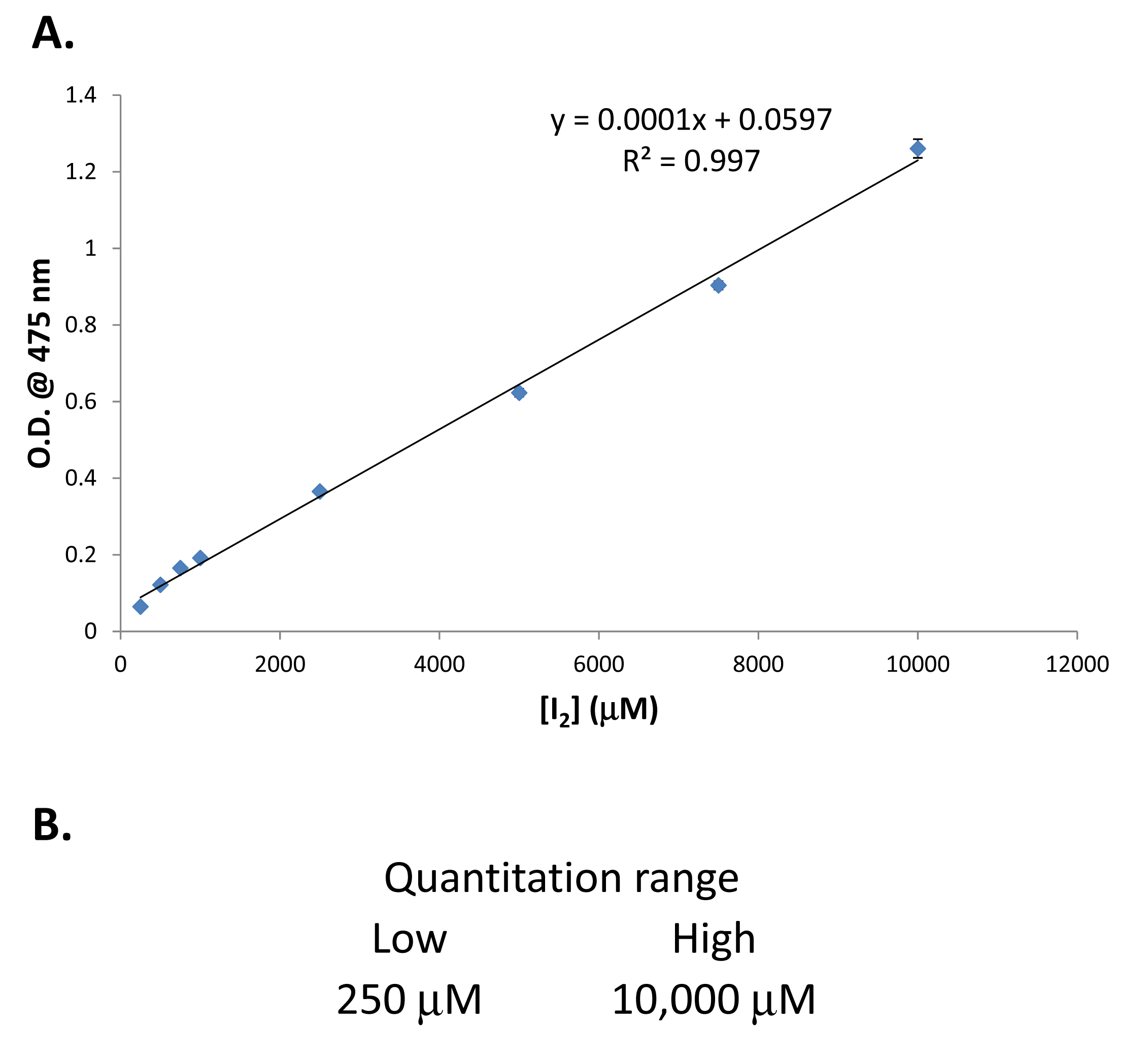

Supplement: Figure S22 — Iodine standard absorbance curve at 475 nm. (TIF) [file pone.0079218.s022.tif]

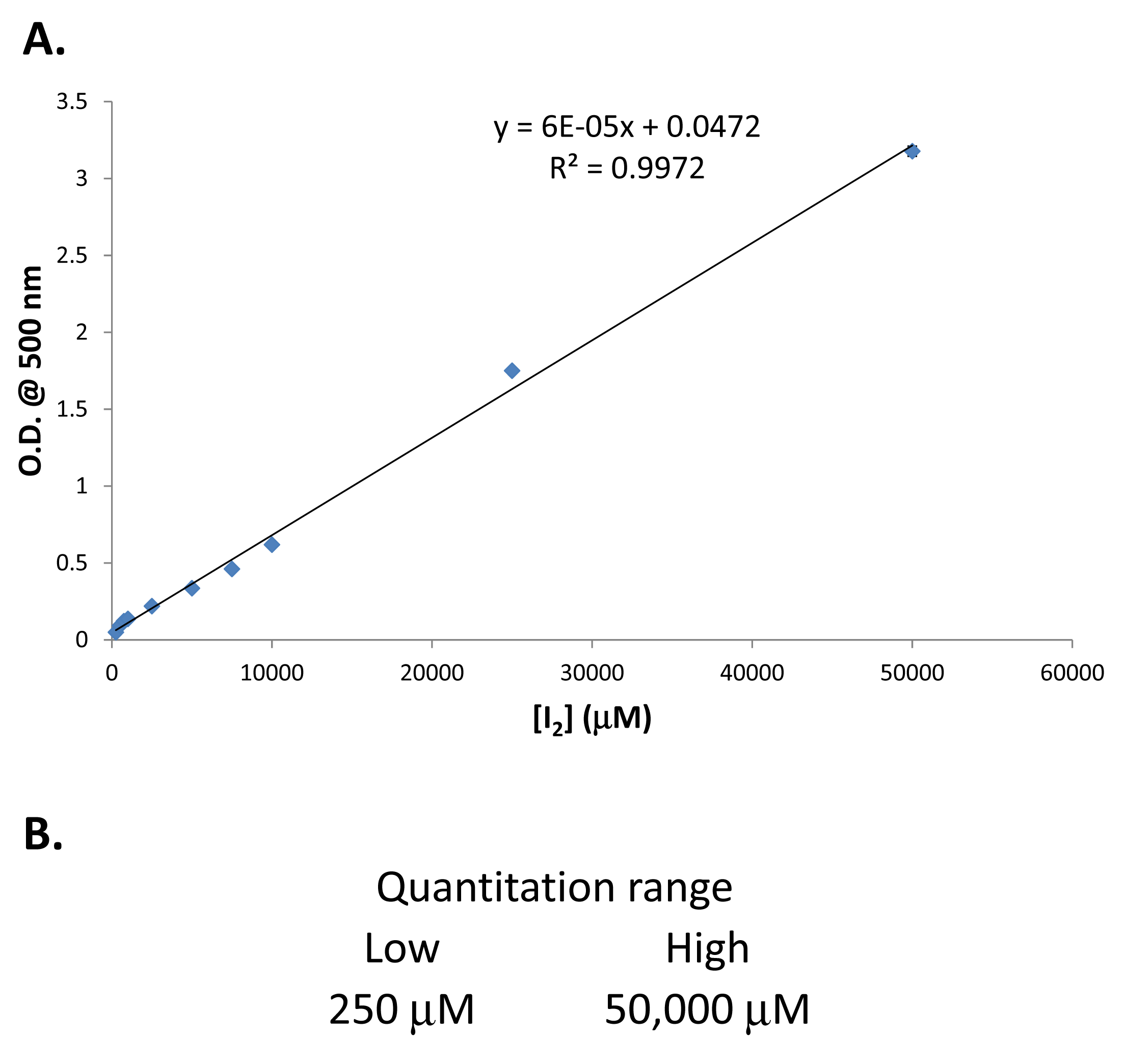

Supplement: Figure S23 — Iodine standard absorbance curve at 500 nm. (TIF) [file pone.0079218.s023.tif]

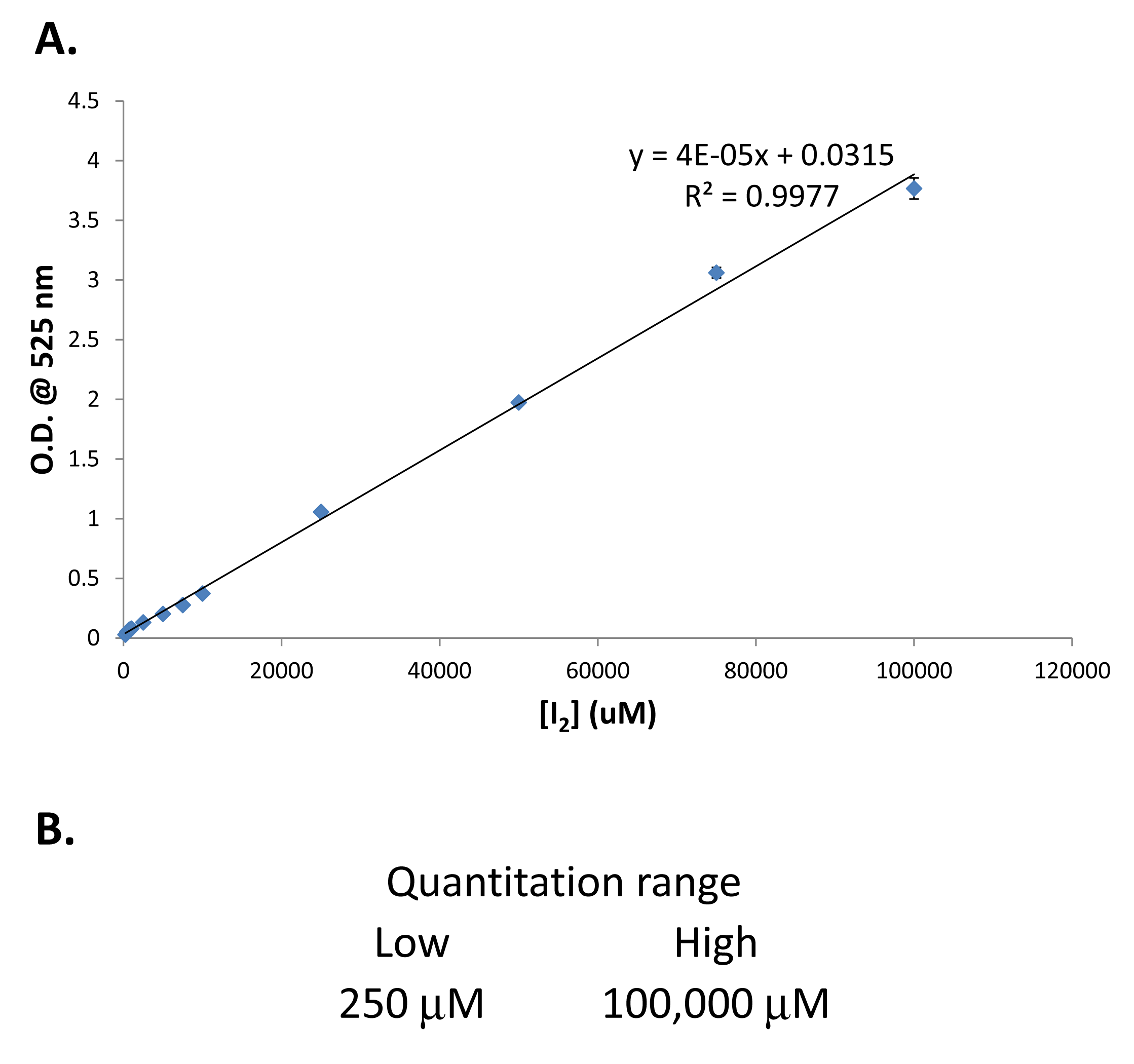

Supplement: Figure S24 — Iodine standard absorbance curve at 525 nm. (TIF) [file pone.0079218.s024.tif]

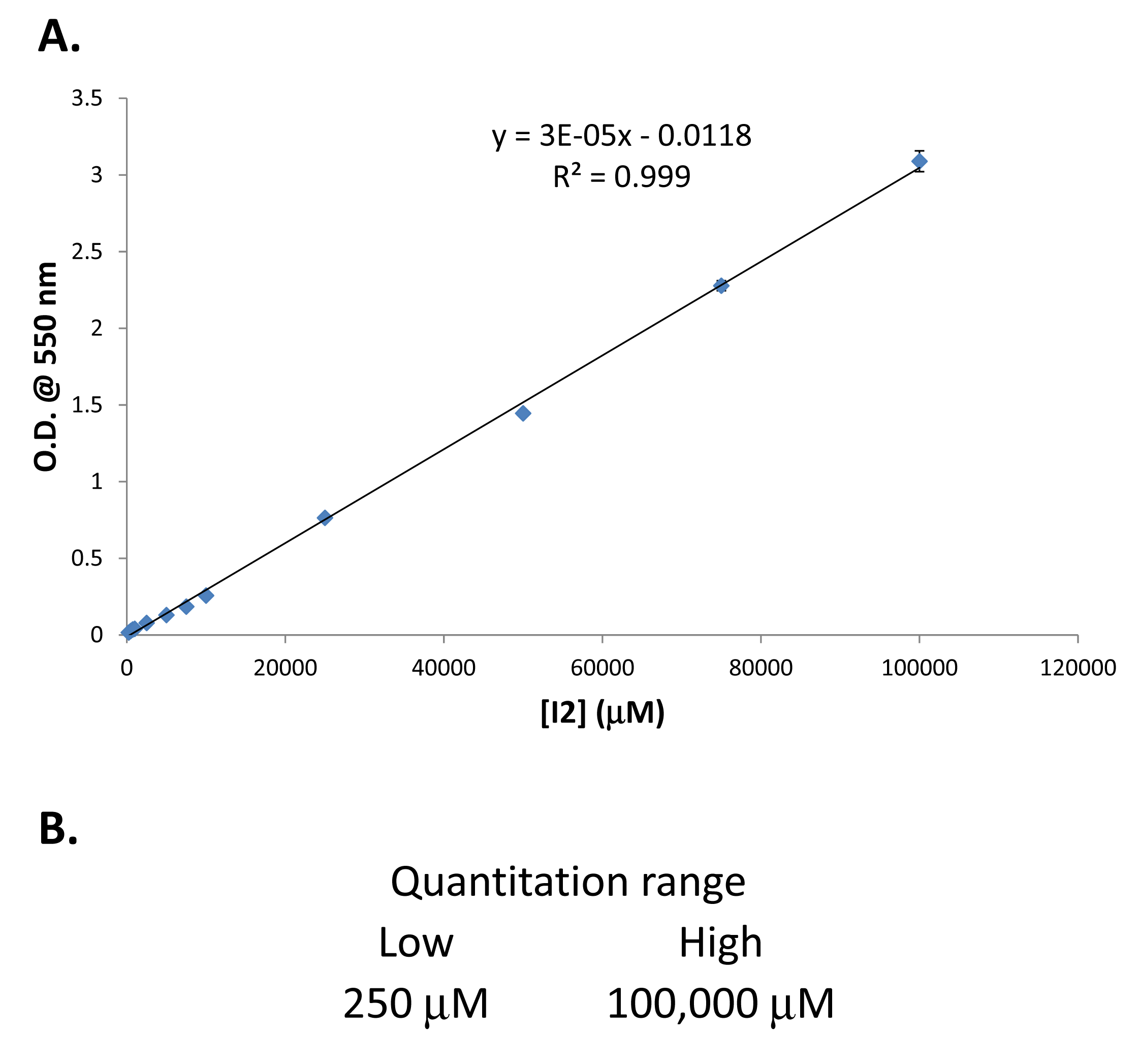

Supplement: Figure S25 — Iodine standard absorbance curve at 550nm. (TIF) [file pone.0079218.s025.tif]

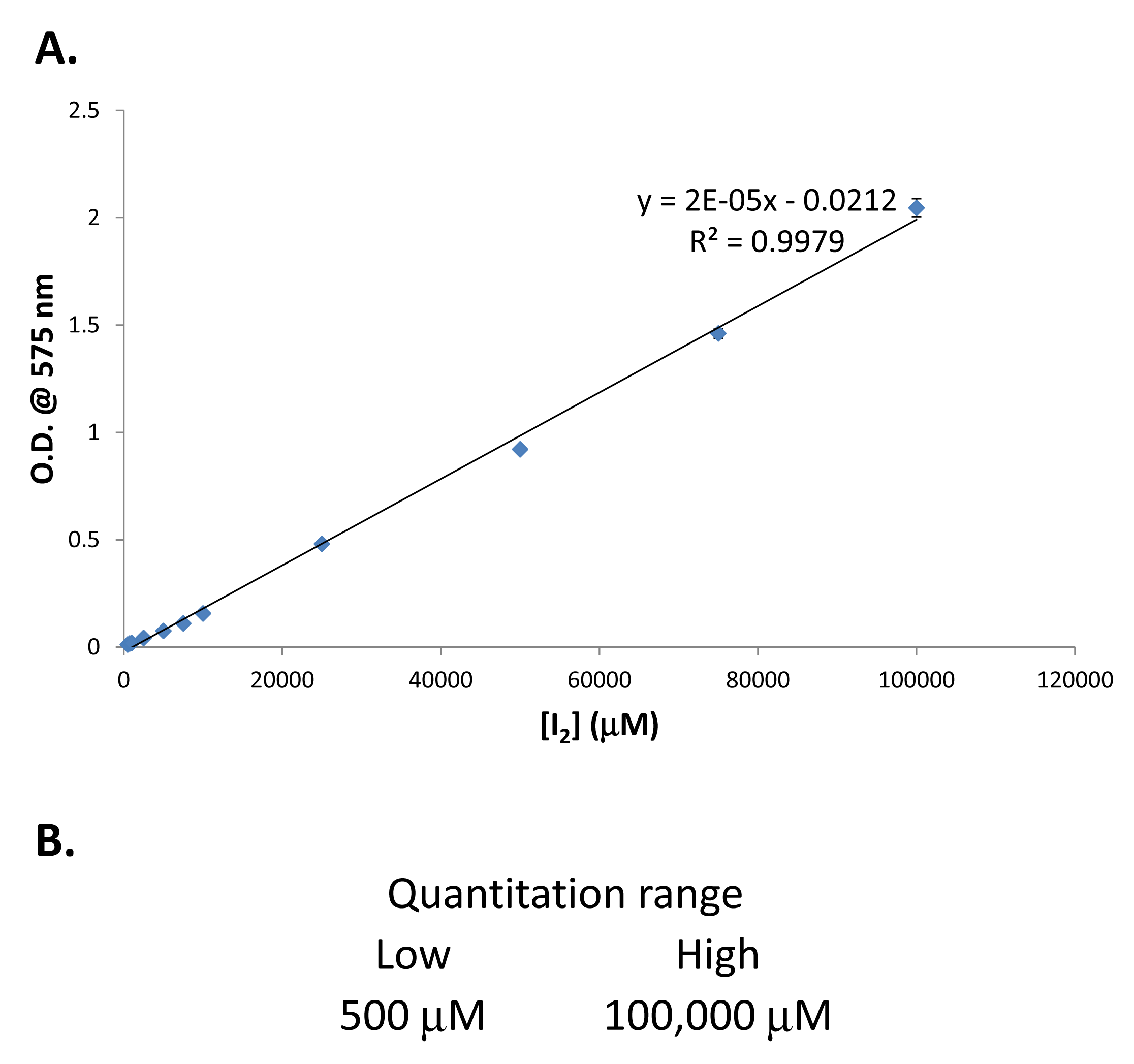

Supplement: Figure S26 — Iodine standard absorbance curve at 575 nm. (TIF) [file pone.0079218.s026.tif]

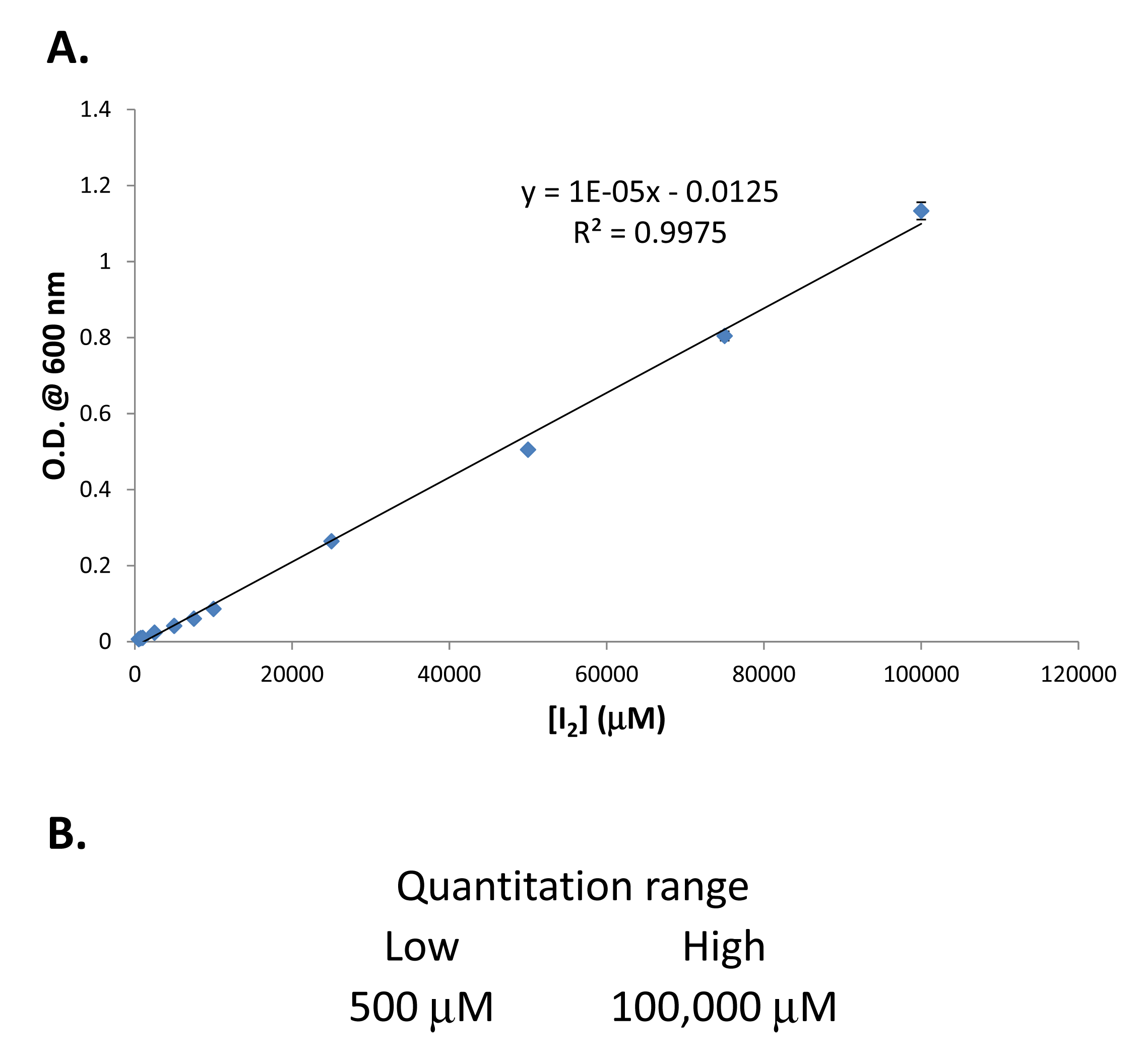

Supplement: Figure S27 — Iodine standard absorbance curve at 600 nm. (TIF) [file pone.0079218.s027.tif]

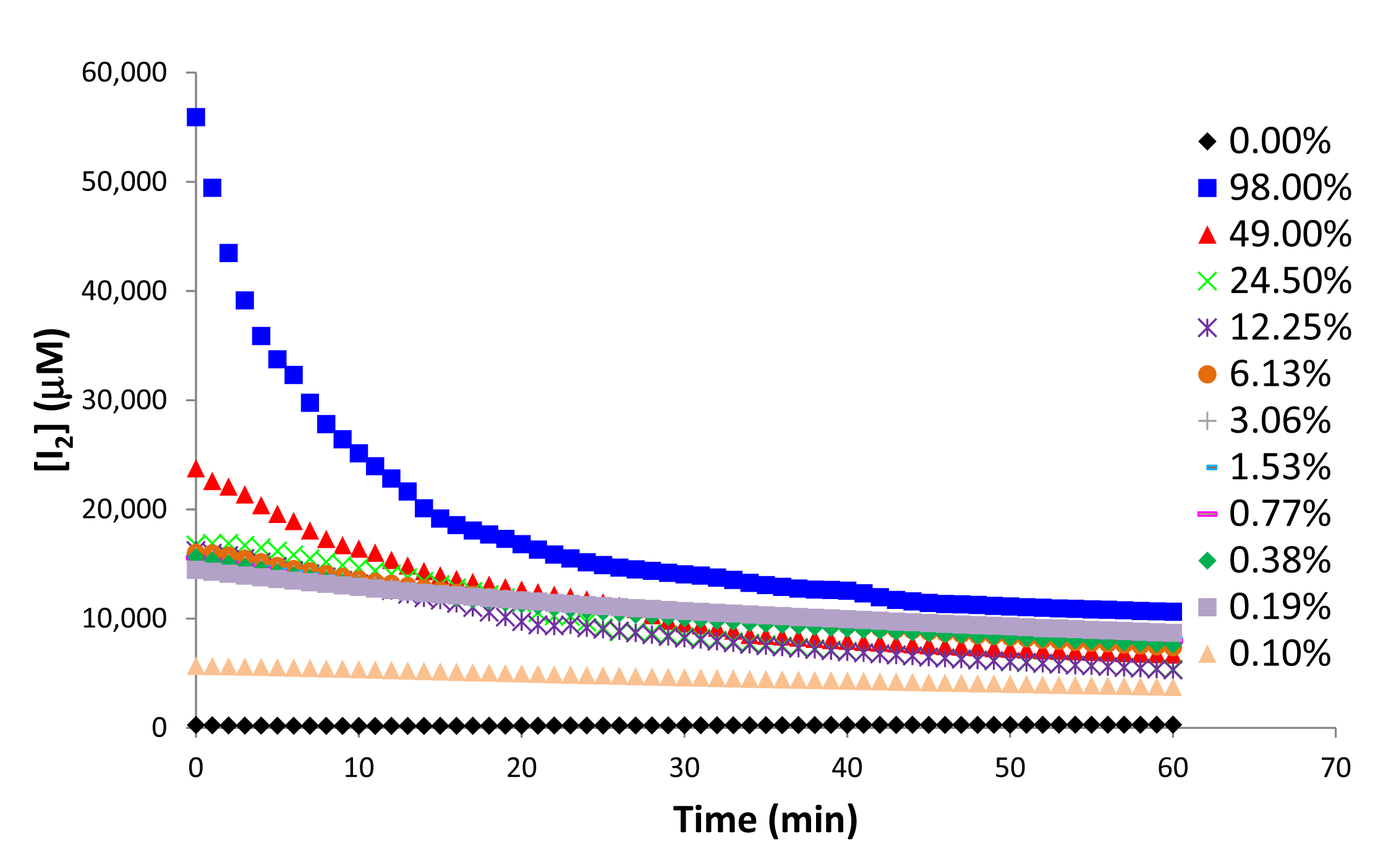

Supplement: Figure S28 — Stability of iodine generated from peroxide – potassium iodide reaction in various sulfuric acid concentrations. (TIF) [file pone.0079218.s028.tif]
